# Mechanism of F_430_-Catalyzed Dehalogenations of Chloromethanes: A DFT Perspective

**DOI:** 10.1021/acs.jpcb.6c00679

**Published:** 2026-04-20

**Authors:** Ye Han, Mateusz Nowicki, Mateusz Pokora, Li Ji, Piotr Paneth

**Affiliations:** † International Center for Research on Innovative Biobased Materials (ICRI-BioM)—International Research Agenda, 49584 Lodz University of Technology, Zeromskiego 116, Lodz 90-924, Poland; ‡ School of Environment and Spatial Informatics, China University of Mining and Technology, Xuzhou 221116, China; ¶ Centre of Molecular and Macromolecular Studies, Polish Academy of Sciences, Sienkiewicza 112, Lodz 90-363, Poland

## Abstract

Rate-determining steps of the alternative mechanisms of reductive dehalogenation of chloromethanes catalyzed by the F_430_ coenzyme and its truncated model have been studied theoretically at the MN15-L/def2-TZVP level of theory which yielded best agreement with the experimental data. Both, explicit and implicit solvent models were used. The energetics of these reactions and structural properties of reactants, products, their complexes, and corresponding transition state have been determined. Based on this information kinetic isotope effects (KIEs) and the corresponding “lambdas” (relation between ^13^C and ^37^Cl KIEs) have been calculated. Two major conclusions come from these studies. First, there is a mechanistic switch with the increasing number of chlorine atoms. Second, for the studied reaction lambdas are not indicative of the mechanism.

## Graphical abstract

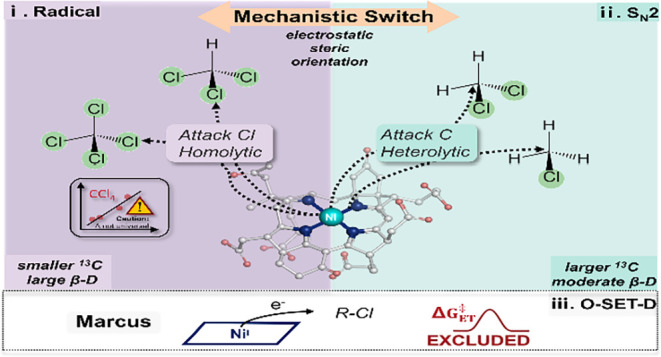

## Introduction

The extensive industrial utilization of halogenated compounds as solvents, chemical intermediates, and pesticides represents a major environmental concern due to their documented adverse impacts on ecosystems and human health.^1^ These detrimental effects are primarily attributed to the presence of halogen substituents; consequently, the transformation of halogenated compounds into less halogenated or nonhalogenated products constitutes a promising strategy for environmental remediation. It is thus not surprising that numerous experimental approaches to dehalogenation, including microbial,^2,3^ enzymatic,^4^ electro-^5^ and photocatalytic,^6^ as well as theoretical studies,^7–9^ have been scrutinized.

Chlorinated alkanes are notorious groundwater contaminants. Their natural reductive dechlorination by microorganisms involves reductive dehalogenases containing cobalamin as a cofactor.^10^ However, underlying general mechanisms of reductive dehalogenation have remained uncertain. Under methanogenic conditions, methanogenic archaea have been demonstrated to degrade trichloroethylene, tetrachloroethylene, and pentachlorophenol in both laboratory experiments and contaminated field sites. In contrast to the majority of anaerobic microorganisms—where vitamin B_12_ typically mediates dehalogenation reactions—the corrinoid F_430_ coenzyme in methanogens has been conclusively shown to play a central role in dehalogenation processes.^11–13^ Coenzyme F_430_ (Figure 1) is the key prosthetic group in methyl-coenzyme M reductase and is essential for methanogenesis in methanogens; it is also implicated in anaerobic oxidation of methane. Hogenkamp et al. investigated the reductive dehalogenation of CH_3_Cl by purified F_430_ isolated from methanogenic archaea, employing titanium­(III) citrate as an electron donor to generate the reduced F_430_–Ni­(I) species in situ.^12^ Their findings showed that the reduction of CH_3_Cl to CH_4_ catalyzed by F_430_–Ni­(I) proceeded at a rate more than 50-fold higher than that observed with the analogous vitamin B_12_–Co­(I) species. Subsequently, Gantzer and Wackett examined the reductive dehalogenation of a range of halogenated organic pollutants catalyzed by several reduced transition metal complexes commonly associated with enzyme active sites in anaerobic microorganisms, including vitamin B_12_, F_430_, and hematin.^14^ Their results further demonstrated that the dehalogenation rates of chloromethanes mediated by F_430_ were substantially higher than those observed with the other two metal coenzymes, whereas F_430_ and B_12_ exhibited comparable reaction rates in the dehalogenation of chloroalkenes. Collectively, these findings provide strong support for the hypothesis that the natural attenuation of halogenated pollutants in anoxic environments is largely dependent on F_430_-containing methanogenic microorganisms. Moreover, the use of purified F_430_, rather than intact microbial cells, for catalyzing dehalogenation reactions offers an effective means of circumventing the toxic effects of high pollutant concentrations on anaerobic microbial communities. However, in contrast to the extensively investigated B_12_-mediated dehalogenation mechanism, only a limited number of studies have addressed the dehalogenation mechanism catalyzed by the F_430_ cofactor to date. Hogenkamp et al. inferred that the higher efficiency of CH_3_Cl dehalogenation by coenzyme F_430_ relative to B_12_ likely arises from the greater lability of the putative carbon–nickel bond in the alkyl–F_430_ intermediate compared with the corresponding carbon–cobalt bond in alkyl corrinoids.^12^ Stolzenberg et al. demonstrated^15^ that a synthetic F_430_ mimic, the nickel­(I) octaethylisobacteriochlorin anion, OEtiBCh-Ni­(I)^−^, reproduces both the structural and functional characteristics of F_430_. Their investigations of the reactions of OEtiBCh with a range of alkyl halides indicated that three pathways—reduction, coupling, and dehydrohalogenation of alkyl halides—can proceed concurrently. These reactions follow second-order kinetics, and increasing the steric bulk of the alkyl substituents of the substrates leads to a decrease in reaction rate. On this basis, Stolzenberg et al. concluded that the Ni­(I) center of OEtiBCh possesses strong nucleophilic character, that formation of alkyl C–Ni intermediates between alkyl halides and Ni­(I) constitutes the rate-determining step, and that the reduction, coupling, and dehalogenation products all derive from these alkyl C–Ni intermediates.^15,16^ In contrast, Castro et al. proposed an S_N_2-type, nonbonding dehalogenation mechanism for the reactions of OEtiBCh with alkyl halides, based on experiments employing free-radical scavengers.^17^ In this mechanism, an electron is transferred from Ni­(I) to the substrate to generate free radicals and halide ions, without the formation of discrete alkyl C–Ni intermediates. Collectively, these findings suggest the existence of multiple, competing reaction pathways for the dehalogenation of halogenated substrates (e.g., chloromethanes, chloroalkenes) mediated by the F_430_ cofactor. The key reactive species, F_430_, is highly transient and can function both as a potent reductant and as a powerful nucleophile. Consequently, resolving the precise dehalogenation mechanism solely through conventional kinetic and analytical techniques remains challenging.

**Figure 1:**
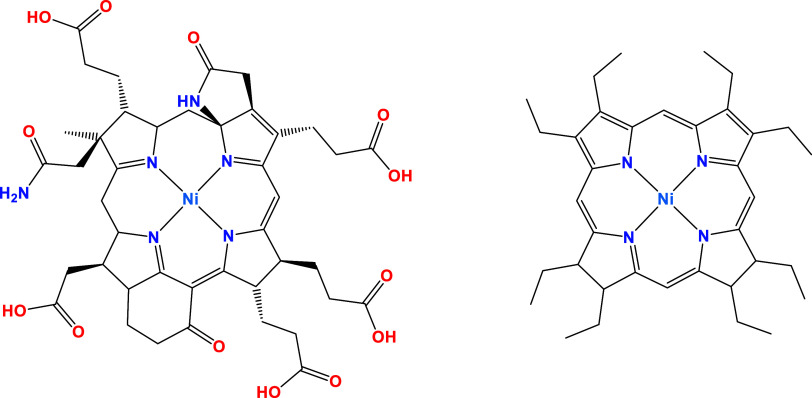
Cofactor F_430_ (left) and octaethylisobacteriochlorin, OEtiBCh (right).

With the development of high-resolution gas chromatography–isotope ratio mass spectrometry (GC-IRMS), compound-specific isotope analysis (CSIA) has become the most widely applied technique in environmental sciences. CSIA quantifies the average isotope enrichment factor of a particular element within a given molecule, and the resulting data are largely unaffected by the presence of other reactants, products, or elements.^18^ Consequently, stable isotope fractionation techniques have been progressively adopted in environmental chemistry. Isotope fractionation refers to the kinetic discrimination between isotopes arising from differences in their physicochemical properties during chemical reactions. The associated isotope effects^19^ reflect differences in the energy profiles between reactants and isotopically substituted transition states. Measured isotope ratios can thus be converted into kinetic isotope effects (KIEs), from which transition state characteristics, and thus mechanistic details can be inferred. In many environmental applications, however, detailed information on the residual substrate fraction and its initial isotopic composition is unavailable or highly uncertain. To address this limitation, a dual-slope (dual-isotope) method has been developed. In this approach, the isotope ratios of two elements are plotted against one another over the course of a degradation or transformation process. This typically yields a linear correlation, the slope of which (the dual-isotope slope, denoted by Λ) represents the relative magnitude of isotope fractionation between the two elements. The method not only facilitates data interpretation but also enables straightforward graphical discrimination among different reaction mechanisms and transformation pathways as illustrated in Figure 2.

**Figure 2:**
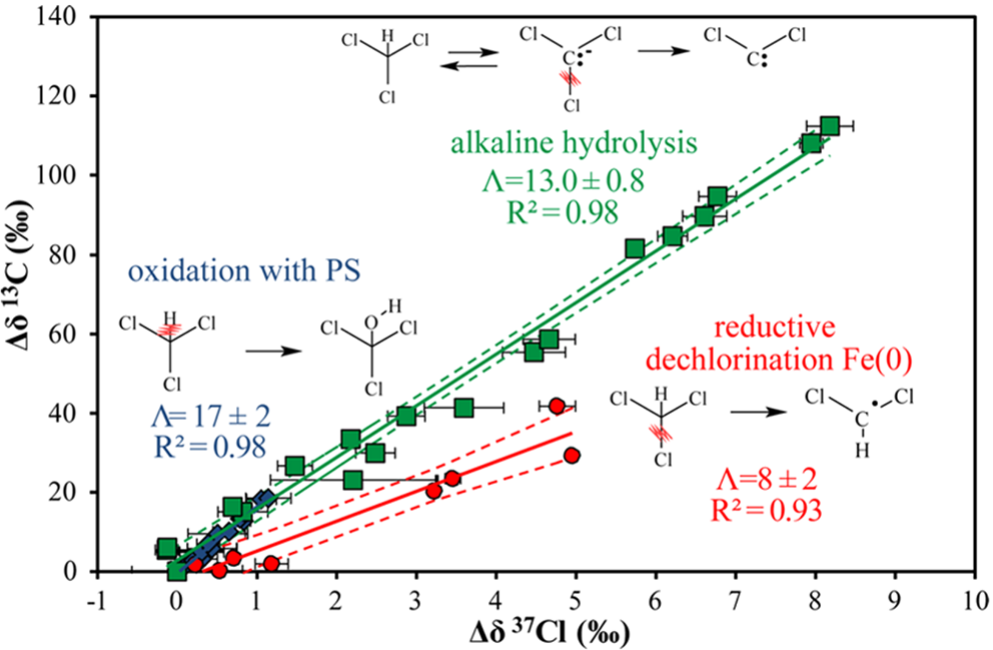
Illustrative C–Cl isotope plot for the three pathways of chloroform degradation. From ref 20 with permission.

In parallel, quantum chemical calculations provide features of transition states along reaction pathways,^21^ which can be used to compute KIEs and, in turn, to derive theoretical isotope fractionation factors. Comparison of these theoretical values with experimentally determined isotope signatures enables the identification of the most plausible reaction mechanisms. Therefore, in recent years, quantum chemical calculations and stable isotope fractionation approaches have assumed increasingly important roles in elucidating biotransformation mechanisms, serving as complementary tools to conventional analytical and kinetic methodologies. In this contribution, we have modeled theoretically dehalogenation of chloromethanes considering different mechanisms illustrated in Scheme 1. We have calculated Gibbs free energies of activation for the rate controlling step and associated chlorine and carbon kinetic isotope effects. We show that there is a switch in the mechanism upon increasing number of chlorine atoms in the molecule. Furthermore, we show that dual-isotope approach is not indicative of the reaction mechanism.

**Scheme 1:**
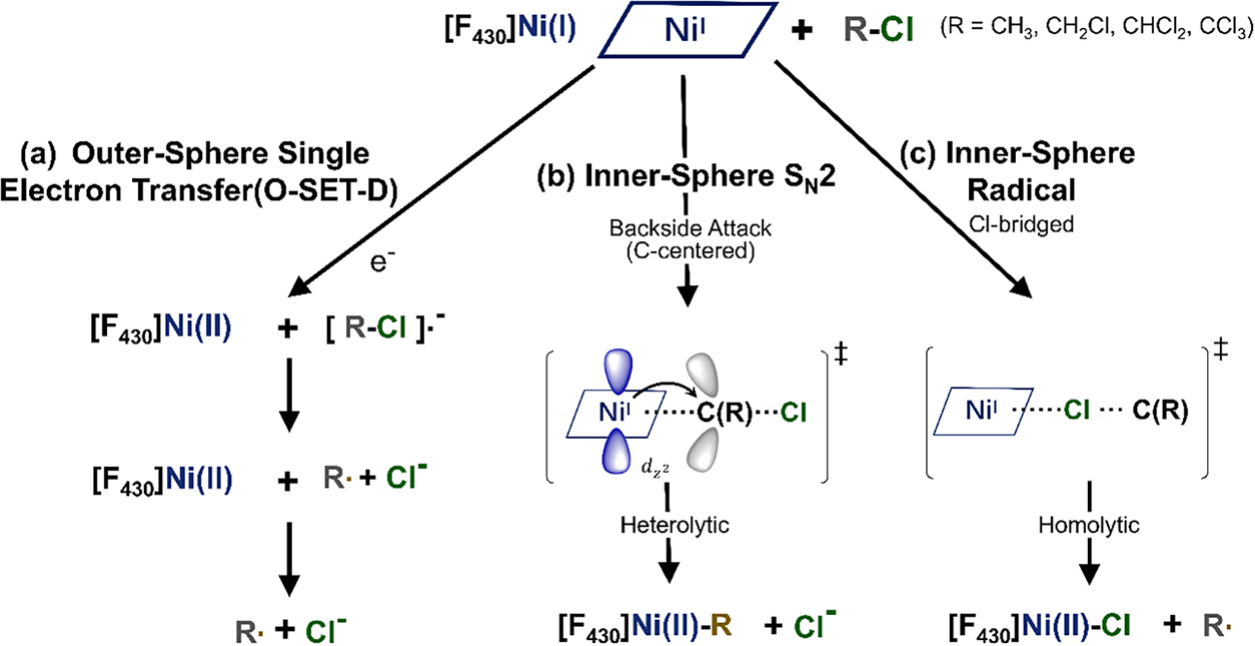
Considered Mechanisms of the Reductive Dichlorination of Chloromethanes by F_430_

## Materials and Methods

### Electronic Structure and Thermochemistry

Following our previous benchmark calculation^9^ all production DFT calculations were performed at the MN15-L/def2-TZVP^22,23^ level with SMD continuum solvation^24^ and DMF as the default solvent. Harmonic frequency analyses at 298 K (without empirical scaling) provided zero-point and thermal corrections and confirmed the nature of all stationary points. All solution-phase free energies are reported at the 1 mol/L standard state. Electrostatic potential charges were obtained according to Merz–Singh–Kollman scheme.^25,26^

### Implicit Solvation

SMD was used throughout. For DMF, the standard SMD parameters embedded in the implementation were employed. For a comparative “soil-like” medium, a dielectric description was used with ε = 15.0 and ε_∞_ = 2.3716; all remaining parameters follow the cited reference for this soil model.^27^

### Explicit DMF Cluster

For the octaethylisobacteriochlorin (OEtiBCh) model, representative C1/TS/C2 microsolvated structures were examined with an explicit shell of DMF. Geometries in the TS region were optimized at five levels: GFN2-xTB^28^ (with and without additional ALPB­(DMF)^29^), MN15-L/def2-SVP:^23^UFF,^30^ MN15-L/def2-TZVP:UFF//MN15-L/def2-SVP:UFF, and MN15-L/def2-TZVP:UFF. Separate, noninteracting substrates and products were not computed for these explicit clusters, because partitioning the solvent sphere would render relative free energies nonmeaningful.

### Conformational Analysis and Ensemble Treatment

For OEtiBCh, we adopted a single scaffold conformer reported previously as the dominant one (following conformational analysis).^9^ Conformers of F_430_ were generated with CREST;^31^ a curated low-energy set of 8 conformers (numbered 1, 2, 5, 10, 17, 20, 23 and 25) was retained for kinetics. One additional conformer (26) was proposed based on conformational search performed using HyperChem,^32^ and another (27) emerged during manual curation of conformer 20. All structures retained for analysis were then fully optimized at the corresponding quantum-chemical level together with the substrate, i.e., neither the F_430_ nor the OEtiBCh scaffold was ever kept frozen.

A detailed conformational analysis (10 conformers) was carried out for CH_3_Cl; based on these results, the 3 kinetically dominant conformers of F_430_ were studied with other substrates. For CH_2_Cl_2_ and CHCl_3_, additional transition state (TS) rotamers were generated by varying the rotation about the C–Cl bond and the trajectory into Ni with both F_430_ and OEtiBCh.

Ensemble observables at 298 K were obtained by Boltzmann weighting over S (separate reactants) free energies

1 $$\Delta G_{B}^{‡}=\sum_{i}w_{i}\left[G_{i}\left(\mathrm{TS}\right)-G_{i}\left(S\right)\right],w_{i}=\frac{e^{-\Delta G_{i}\left(S\right)/\mathrm{RT}}}{\sum_{j}e^{-\Delta G_{j}\left(S\right)/\mathrm{RT}}}$$

where Δ*G_i_* (TS – S) = Δ*G_i_* (TS) – Δ*G_i_* (S) for conformer *i*. Eyring-weighted checks were also performed

2 $$\Delta G_{E}^{‡}=-\mathrm{RT}\ln\left(k_{\mathrm{eff}}\right),k_{\mathrm{eff}}=\sum_{i}e^{-\Delta G_{i}^{‡}/\mathrm{RT}}$$

with Δ*G*_i_^‡^ = Δ*G_i_* (TS) – Δ*G*_min_(S). Whenever geometric or electronic parameters are given, they are derived from the pathway with the lowest Δ*G* ^‡^ on the global S reference surface.

### Outer Electron Transfer Calculation

The Marcus–Hush theory calculations employed Savéant’s model, as shown in eqs 3 and 4. The reaction Gibbs free energy Δ*G*_ET_^‡^ related to the inner reorganization energy (λ_*i*_), the external reorganization energy λ_O_, and the intrinsic barrier Δ*G*_ET_. In the intrinsic barrier Δ*G*_ET_ of this system, we considered bond dissociation Gibbs free energy Δ*G*_C–X_. The external reorganization energy λ_0_ can be calculated using eq 5.^33,34^

3 $$\Delta G_{\mathrm{ET}}^{‡}=\frac{\lambda }{4}{\left(1+\frac{\Delta G_{\mathrm{ET}}}{\lambda }\right)}^{2}$$

4 $$\lambda =\lambda_{O}+\lambda_{i}+{\Delta G}_{C\text{−}X}$$

5 $$\lambda_{0}=\frac{N_{A}e^{2}}{4\pi \varepsilon_{0}}\left(\frac{1}{\varepsilon_{\mathrm{op}}}-\frac{1}{\varepsilon_{s}}\right)\left(\frac{1}{2r_{1}}+\frac{1}{2r_{2}}-\frac{1}{R}\right)$$

### Geometries, Transition States, and Validation

All stationary points were optimized at the level stated for each data set with tight thresholds and ultrafine integration grids. Transition states were located with standard algorithms and validated by intrinsic reaction coordinate (IRC)^35–41^ calculations in both directions, connecting C1 and C2 for the same mechanism. A single imaginary frequency was confirmed for each TS; tabulated TS descriptors are reported in the Results/SI where relevant.

### Kinetic Isotope Effects (KIE)

Primary ^13^C (reaction center), ^37^Cl (leaving group), and secondary ^37^Cl and ^2^H (β-D) KIEs were computed at 298 K via the Bigeleisen–Mayer formalism^42–44^ using unscaled harmonic frequencies at the optimization level. Two referencing conventions were evaluated–relative to S (isolated substrates) and relative to C1 (encounter complex); the resulting KIEs are close to each other (typically ±0.1% for ^37^Cl and ±0.2% for ^13^C), so only the former values are reported in the main text; for full data, please refer to Tables S13–S14 in Supporting Information (SI). Λ­(C/Cl) is calculated according to eq 6.

6 $$\Lambda \left(\frac{C}{\mathrm{Cl}}\right)=\frac{\frac{1}{{\mathrm{KIE}}_{C}}-1}{\frac{1}{{\mathrm{KIE}}_{\mathrm{Cl}}}-1}$$

### Software

Gaussian 16 ^45^ was used for all DFT and DFT:UFF optimizations, frequencies, and thermochemistry; ORCA 5 ^46–48^ was used for GFN2-xTB^49^ calculations on explicit DMF clusters. CREST^50^ was used for conformational searches. ISOEFF17 ^51^ was used for KIE calculations. ChemDraw Professional 16, VMD^52^ and Energy Diagram Plotter^53^ were used for generating figures and schemes.

## Results and Discussion

Our major goal was to study mechanisms of dehalogenation of chloromethanes catalyzed by the F_430_ coenzyme. We have used MN15-L/def2-TZVP level of theory which showed best agreement with the experimental results.^9^ For comparison with our previous benchmark studies we have in parallel carried out calculations for its truncated OEtiBCh model. Since, however, obtained results for both catalysts are qualitatively (and nearly quantitatively) the same for the clarity of the presentation the results obtained for OEtiBCh are only in the Supporting Information.

### Conformational Analysis

We first quantified how conformational degrees of freedom shape the free-energy landscape and whether explicit ensemble averaging is necessary for reliable barriers. Two robust conclusions emerge from this analysis: (i) The global minimum at S controls the effective barrier (Figure 3). In the S_N_2 case F_430_ + CH_3_Cl, conformer 27 is the global minimum at S and its complex with CH_3_Cl gives the most stable configuration of C1. Its intrinsic barrier Δ*G*(TS – S) is not the lowest in the set–Interestingly, conformer 1 offers a slightly lower intrinsic barrier Δ*G*(TS_1_ – S_1_); however, as Gibbs free energy of conformer 1 is 2.3 kcal/mol above the global minimum at S, and its overall barrier Δ*G*(TS_1_ – S_27_) is therefore higher. Thus, the conformer that dominates the equilibrium at S need not minimize the intrinsic barrier, but it does determine the observed Δ*G* ^‡^ because other conformers are less stable at S. Ensemble weighting confirms this: both the Boltzmann-weighted and Eyring-weighted barriers are essentially identical to the barrier of conformer 27 alone (Table S1). These findings emphasizes the importance of the conformer search, as without conformer 27 the reported Δ*G* ^‡^ would be lower by ca. 1 kcal/mol.

**Figure 3:**
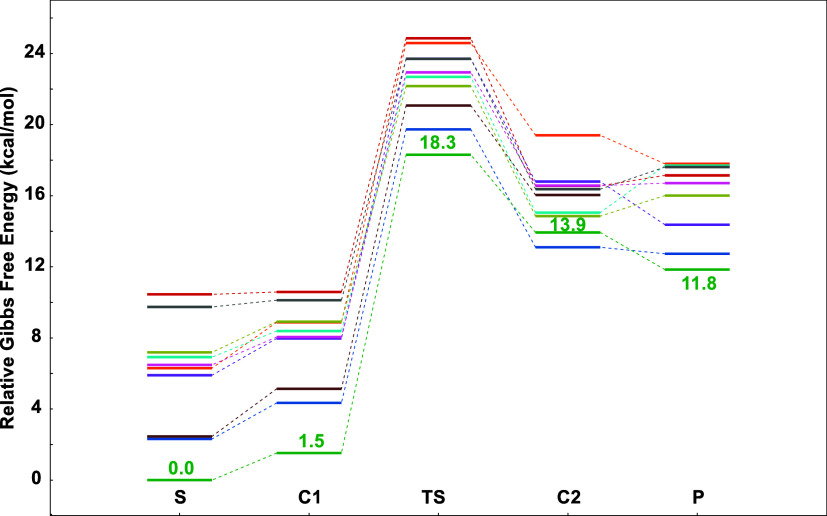
S_N_2 dehalogenation of CH_3_Cl on F_430_: Gibbs free energy profiles for the 10 conformers with lowest Δ*G* ^‡^. Numerical values are given for the preferred conformer.

(ii) “Best-conformer ≈ ensemble” generalizes across mechanisms and substrates. We verified this conclusion beyond the CH_3_Cl S_N_2 test. Along the radical pathway with F_430_, families of macrocycle conformers and substrate orientations were evaluated. For each substrate (CH_3_Cl, CH_2_Cl_2_, CHCl_3_, CCl_4_), ensemble-weighted Δ*G* ^‡^ typically differs from the lowest ΔG^‡^ for individual conformer by not more than 0.1 kcal/mol. In practice, the dominant S conformer–and, for OEtiBCh, the physically reasonable approach geometry–governs the barrier; explicit ensemble averaging validates but does not materially change the picture.

These points justify the convention used here: we report energies for the kinetically dominant conformer of the catalyst (the global-minimum S) and, within that, the lowest-energy, sterically and electronically sensible substrate approach (the minimum C1).

Beyond numerics, the analysis is mechanistically instructive. Conformational control is exerted primarily in the reactant basin: the internal flexibility of F_430_ determines which substrate-binding pose is populated at equilibrium; once that pose is fixed, the path to TS is comparatively stereotyped. Conversely, intrinsic barriers Δ*G*(TS – S) taken from an arbitrarily chosen conformer can be misleading if that conformer is not competitive at S; only barriers referenced to the global S (or proper ensemble averages) are mechanistically meaningful.

### Solvent Effects and the Fate of the Explicit-Solvent Model

We next analyzed how solvation modulates barriers and thermodynamics, both to decide which solvation protocol to carry forward and to understand qualitative polarity trends. In addition to implicit DMF, two complementary tests were performed: (i) an explicit DMF cluster around the S_N_2 reaction system, evaluated at several levels of theory (Figure 4), and (ii) implicit-solvent scans comparing DMF, water, and a soil-like dielectric.

**Figure 4:**
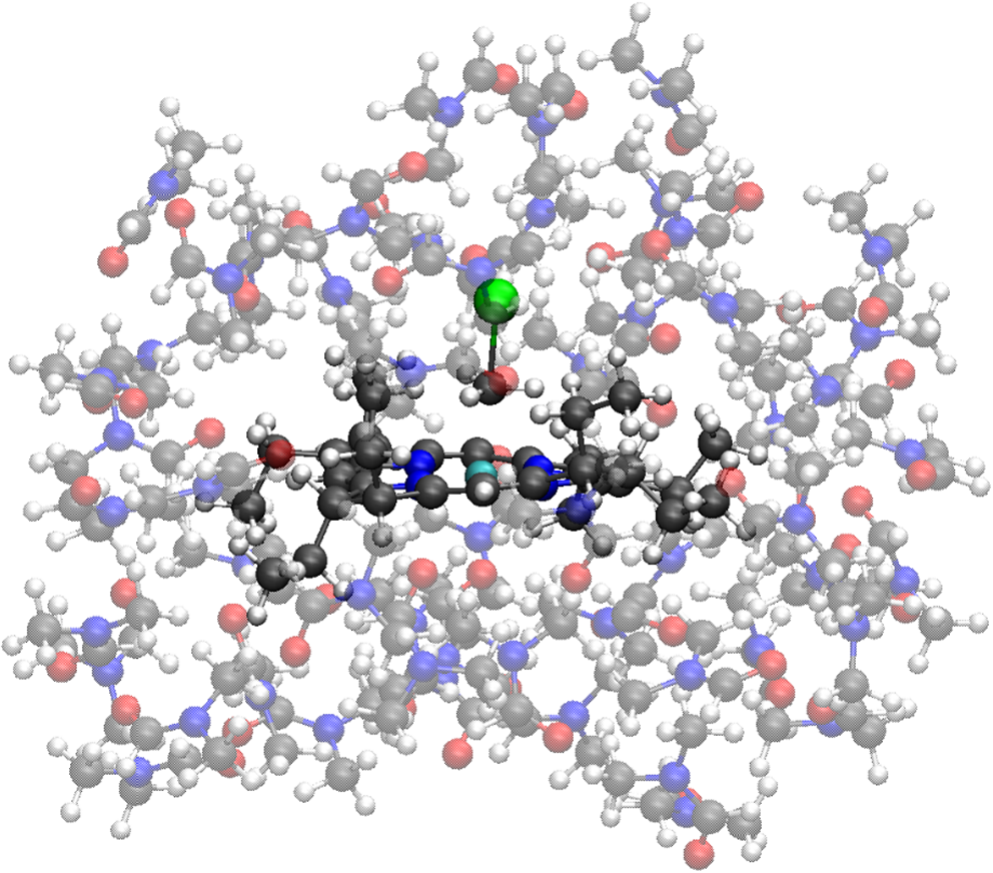
S_N_2 dehalogenation of CH_3_Cl on OEtiBCh in explicit DMF cluster: Transition state structure (Ni–cyan; C–gray; H–white; O–red; N–blue; Cl–green).

Explicit-solvent calculations were confined to the inner portion of the reaction coordinate–C1 → TS → C2–because partitioning a single DMF cluster into two nonoverlapping solvation spheres for separated substrates/products is arbitrary and prone to artifacts. Across methods (Figure S1 and Table S2) the TS–C1 barriers of OEtiBCh + CH_3_Cl reaction cluster near 14–18 kcal/mol. Two features are consistent. First, systematic overestimation versus experiment: the experimental activation free energy in DMF for the simplified system is ca. 12 kcal/mol, yet the explicit-cluster barriers are higher by ca. 2–7 kcal/mol even though they are referenced to C1. Because our implicit-solvent surfaces place S below C1, referencing to S would raise the explicit-cluster Δ*G* ^‡^ further. Second, method-independence of the failure mode: while absolute numbers vary modestly, the qualitative mismatch persists. Technical attempts to broaden sampling either failed to converge or were prohibitive. For F_430_, trial optimizations with an explicit DMF shell relaxed back to substrates even when initialized near products, underscoring that finite clusters cannot stably represent the ion-pair basin for this highly polar step.

Given these points, and because finite clusters require ad hoc choices of size, composition and truncating the long-range dielectric response essential in polar media, we did not pursue the explicit-cluster model for quantitative comparisons. It remains useful as a check that the C1 → TS → C2 topology is reasonable, but it overestimates barriers and introduces uncontrolled boundary errors.

The SMD treatment, on the other hand, provides a clean, reproducible reference applicable across substrates and mechanisms. For F_430_ + CH_3_Cl (S_N_2 manifold), moving from water to DMF and to a lower-polarity soil proxy produces modest, monotonic shifts in barrier height (Figure 5 and Table S3). This pattern is chemically transparent. The S_N_2-like TS involves partial C–Cl cleavage and growing charge separation that benefit from a polar medium; reducing the bulk dielectric penalizes both the TS and the post-TS ion-pair well (C2). Crucially, the magnitude of the solvent effect is modest (ca. 1–2 kcal/mol for CH_3_Cl), so relative mechanistic trends (S_N_2 vs radical across substrates) remain intact when we use DMF. For the simplified OEtiBCh model, DMF is also the medium where experimental kinetics are available and where implicit surfaces reproduce those kinetics far better than the explicit-cluster alternative. For F_430_, adopting DMF aligns with the planned experiments and enables direct comparison to OEtiBCh.

**Figure 5:**
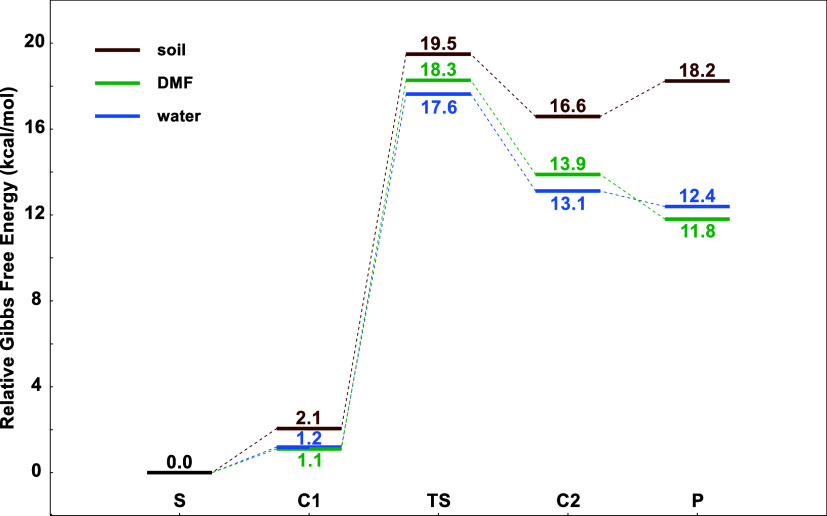
S_N_2 dehalogenation of CH_3_Cl on: Gibbs free energy profiles in different media modeled implicitly (SMD).

### The S_N_2 Manifold: How Substrate Chlorination and Pocket Architecture Sculpt the TS

We first analyze the S_N_2 surface for F_430_ in implicit DMF, using two complementary views: (i) Gibbs free-energy profiles (Figure 6 and Table S4) and (ii) TS descriptors–the magnitude of the imaginary frequency |ν_im_|, the forming Ni–C (or Ni–Cl) distance, the breaking C–Cl distance, and the approach angle at carbon (Ni–C–Cl or Ni–Cl–C; Figures 7 and 8 and Table S6). Together they reveal a continuous progression from classical backside substitution for CH_3_Cl toward a halogen-bridged, quasi-dissociative S_N_2 limit as chlorination increases–an evolution accentuated by the steric and electrostatic field of the full F_430_ pocket.

**Figure 6:**
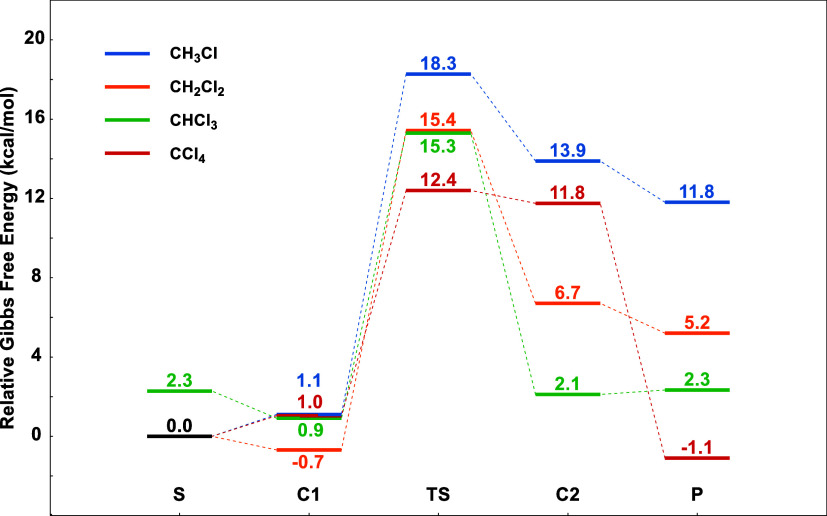
S_N_2 dehalogenation of chloromethanes on F_430_: Gibbs free energy profiles.

**Figure 7:**
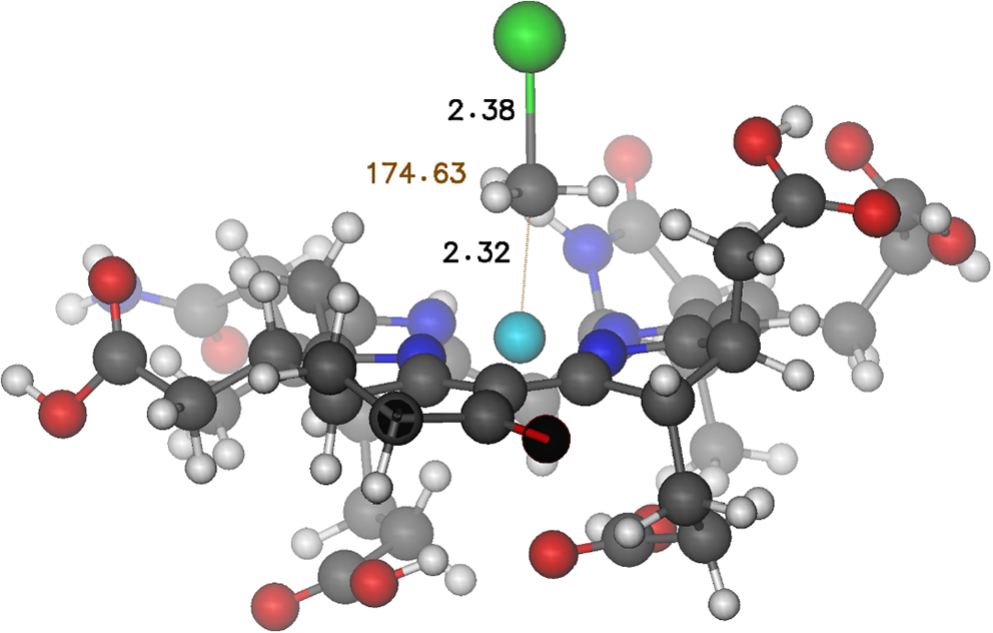
S_N_2 dehalogenation of CH_3_Cl on F_430_: Transition state structure.

**Figure 8:**
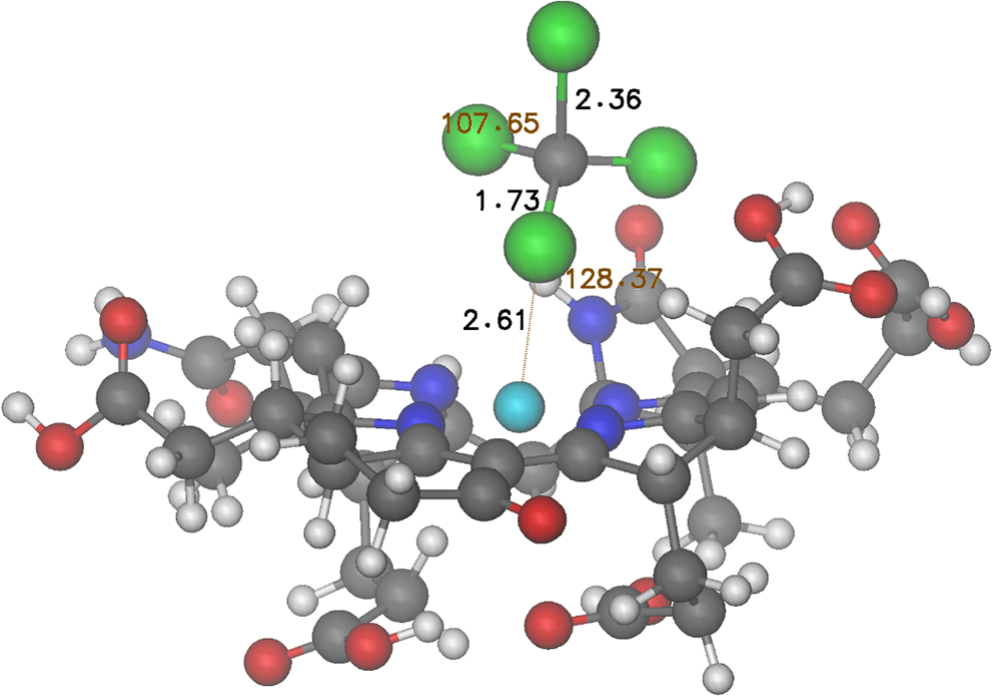
S_N_2 dehalogenation of CCl_4_ on F_430_: Transition state structure.

The S_N_2 barrier decreases monotonically with chlorination (CH_3_Cl → CH_2_Cl_2_ → CHCl_3_ → CCl_4_). The postreaction complex (C2) and products become generally more stabilized, consistent with easier chloride departure and a softer, more polarizable electrophile; CCl_4_ breaks the trend with C2 well and the products being destabilized (C2 lies up to 12 kcal/mol above S; Figure 6).

The geometric metrics rationalize the energetics (Table S6). In F_430_ the forming Ni–C distance increases markedly with chlorination, from 2.32 Å (CH_3_Cl) to 3.93 Å (CCl_4_), while the approach angle evolves from near-linear (175°) to bent for CH_2_Cl_2_/CHCl_3_/CCl_4_ (149°, 136° and 139°, respectively). The breaking C–Cl bond is only modestly elongated across the series (2.36–2.43 Å). Concomitantly, |ν_im_| decreases from the mid-300s cm^–1^ (CH_3_Cl/CH_2_Cl_2_) to 295 cm^–1^ (CHCl_3_) and 161 cm^–1^ (CCl_4_), indicating a geometrically extended reaction coordinate. In the case of CCl_4_, one of the nonleaving chlorine atoms can be clearly noticed to bridge the nickel atom and the carbon atom of CCl_4_ (2.61 Å). This, together with the very long Ni–C and relatively shorter C–Cl at the CCl_4_ TS make the assistance character explicit: the nickel center primarily facilitates heterolysis rather than forming a compact, three-center TS. A practical corollary is that CCl_4_ never permits a tight Ni–C approach inside the cofactor. The TS is far on the dissociative side (Ni–C = ∼3.93 Å) and the C–Ni distance in C2 (3.68 Å) remains close to TS values, reinforcing this topology.

### The Radical Manifold: Ni–Cl Capture and Carbon-Radical Release

On the radical surface the catalyst assists homolytic C–Cl cleavage while forming Ni–Cl; the carbon departs as R^•^. In implicit DMF this pathway strengthens with increasing chlorination and, for the most chlorinated substrates, overtakes S_N_2. Below we discuss energetics (Figure 9 and Table S7) and TS structure (Figures 10 and 11 and Table S9), before comparing to S_N_2.

**Figure 9:**
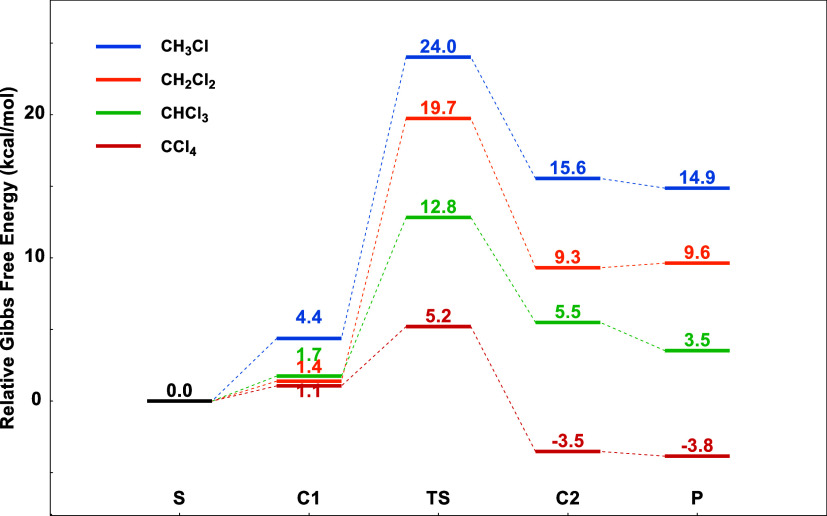
Radical dehalogenation of chloromethanes on F_430_: Gibbs free energy profiles.

**Figure 10:**
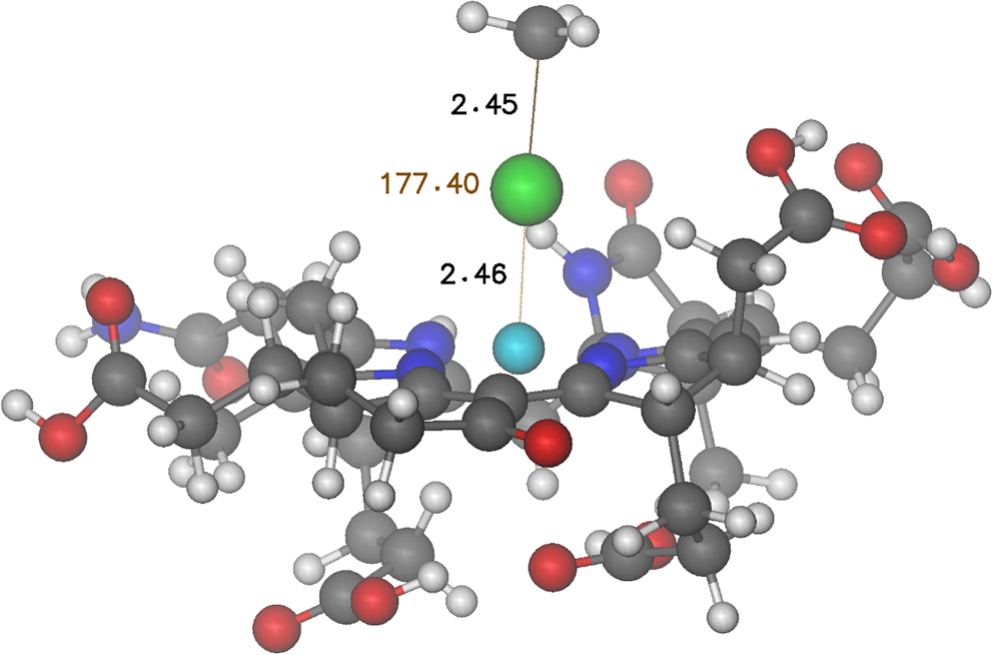
Radical dehalogenation of CH_3_Cl on F_430_: Transition state structure.

**Figure 11:**
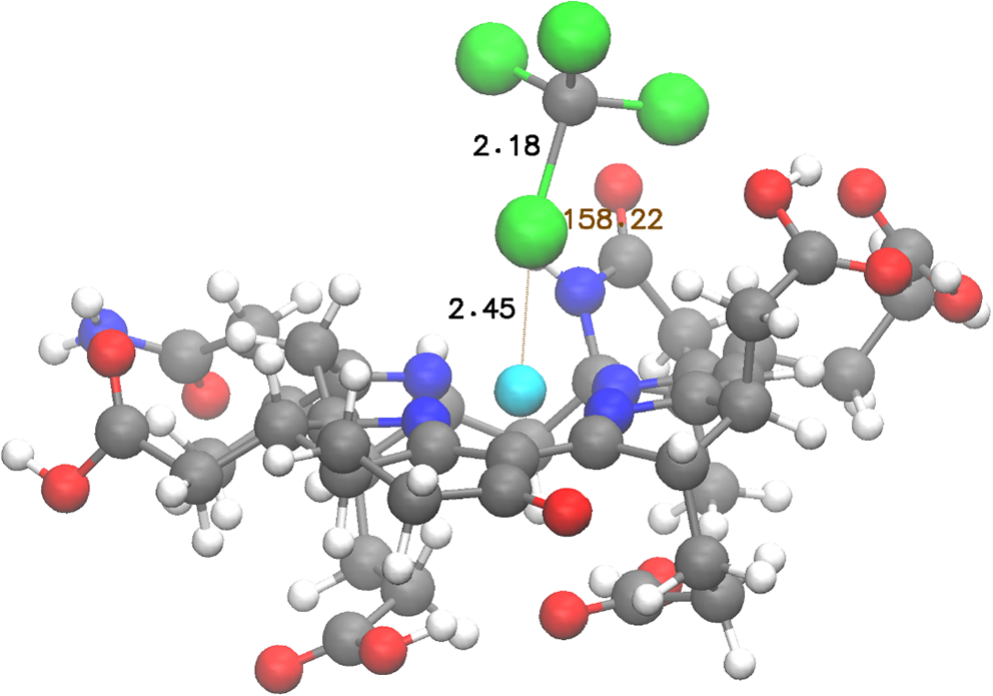
Radical dehalogenation of CCl_4_ on F_430_: Transition state structure.

The radical Δ*G* ^‡^ falls with chlorination: large for CH_3_Cl, moderate for CH_2_Cl_2_, and low for CHCl_3_ and CCl_4_; the corresponding products evolve from highly endergonic (CH_3_Cl) to slightly exergonic (CCl_4_) (Figure 9). Thus, the radical pathway is noncompetitive for CH_3_Cl and CH_2_Cl_2_, but favored for CHCl_3_ and CCl_4_. A broader conformational survey confirms that Boltzmann-weighted barriers are essentially identical to the lowest ΔG^‡^ value for individual conformer (and Ni–C–Cl rotamer) in each case (Table S7). The mean Δ*G* ^‡^ decreases monotonically across the series, mirroring the growing driving force for Ni–Cl formation and radical stabilization.

ESP charges strongly support this picture of distinct charge-separation patterns for S_N_2 and radical pathways on F_430_ (Tables S10–S11). For S_N_2, the MK charges on the haloalkane fragment show substantial cationic character developing at carbon in the TS and then relaxing as chloride becomes strongly anionic in C2. For example, in CH_3_Cl the −CH_3_ fragment carries a charge of about +0.19 e and the leaving Cl about −0.64 e in the TS, evolving to −0.04 e on carbon and −0.89 e on Cl in C2. In contrast, along the radical pathway the −R fragment remains close to neutral or slightly negative and no pronounced cationic build-up at carbon is observed; the main change is the growth of negative charge on the Ni-bound chloride. This pattern is fully consistent with a heterolytic, ion-pair-like S_N_2 coordinate versus a homolytic Ni–Cl capture for the radical pathway.

TS descriptors paint a coherent picture of a compact chloride-transfer event whose character shifts earlier along C–Cl as the reaction becomes more exergonic. The forming Ni–Cl distance is short and nearly invariant (2.45–2.46 Å). The C–Cl bond in the TS becomes progressively less elongated with chlorination (from 2.40 Å for CH_3_Cl to 2.29 Å for CHCl_3_ and 2.18 Å for CCl_4_), consistent with an earlier TS as Δ*G* becomes more negative. The approach angle hovers near 154–177°, indicating a bent but directed approach of Ni toward the leaving chloride; unlike S_N_2, backside attack at carbon is not required. The magnitude of the imaginary frequency |ν_im_| decreases with chlorination (from 374 cm^–1^ in CH_3_Cl to 242 cm^–1^ in CCl_4_), reflecting a looser coordinate as the surface steepens toward products. Together, these metrics confirm a Ni–Cl-localized TS whose “earliness” increases with halogenation.

The radical TS is governed by the macrocycle conformer chosen and by how the substrate is rotated about the R–C–Cl bond when the substrate symmetry is reduced. In CH_2_Cl_2_ and CHCl_3_, twisting this dihedral modulates the alignment between the Ni approach vector and σ*­(C–Cl). Good alignment produces an earlier, tighter TS and a noticeably lower barrier; misalignment yields a looser TS and a penalty on the order of 1 kcal/mol under the same F_430_ conformer. In contrast, CH_3_Cl (nearly isotropic σ-framework) and CCl_4_ (high symmetry) show minimal sensitivity to this rotation: the same Ni–Cl-forming motif is accessible over a broad range of torsional angles, and the barrier is largely determined by pocket packing rather than substrate torsion. When conformers are Boltzmann-weighted relative to S, ensemble-averaged barriers track the best-aligned rotamers closely; thus the main text reports only the lowest Δ*G* ^‡^ values for each reaction, with full rotamer analysis in the Supporting Information.

### Dissociative Outer-Sphere Single Electron Transfer (O-SET-D)

While the S_N_2 and radical reaction pathways can account for most of the observed reactivity, theoretically, dissociative- outer-shell electron transfer (O-SET-D) could also compete for these benefits in highly polar media. Since both OEtiBCh and F_430_ function in polar environments such as DMF, aqueous phases, and soil matrices, the high dielectric constant of these media can stabilize charge-separated intermediates. Therefore, to evaluate whether such an effect could render the O-SET pathway viable, we explicitly examined the dissociative outer-sphere single-electron transfer (O-SET-D) mechanism. Similar long-range charge-transfer mechanisms have been proposed for low-valent metal catalysts, where solvent polarity or redox potential may favor outer-sphere rather than inner-sphere or covalent bond-forming processes.^54,55^

To assess this possibility, we constructed hypothetical O-SET-D reaction profiles using the same series of chlorinated substrates (CH_3_Cl, CH_2_Cl_2_, CHCl_3_, and CCl_4_). The proposed mechanism is illustrated in Scheme 2, where the Ni­(I) species of OEtiBCh or F_430_ donates an electron to the antibonding σ* of R–X, generating a transient [R–X]^•–^ radical anion, which rapidly dissociates to yield R^•^ and X.

**Scheme 2:**
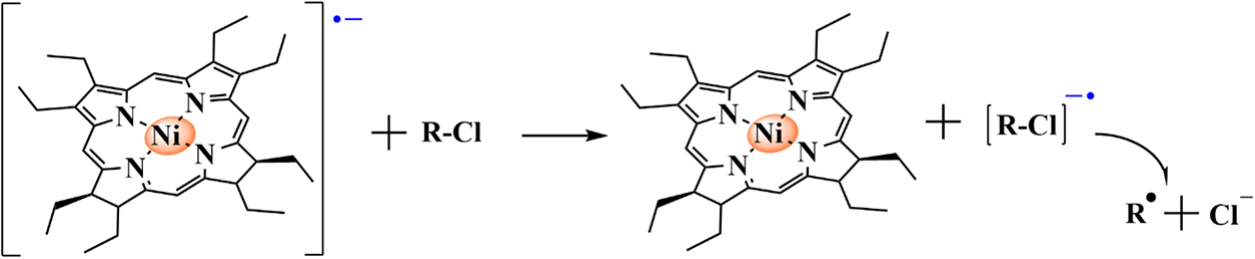
Reaction of OEtiBCh with R–Cl May Involve an Outer Sphere Dissociation Single-Electron Transfer Pathway

Since a stable [R–X]^•–^ intermediate could not be located computationally, its presence was inferred as a short-lived transition configuration consistent with a dissociative electron transfer process.

The free energies of four representative “sticky” outer-sphere electron transfer (O-SET-D) reactions were calculated using the Marcus–Hush framework in conjunction with Savéant’s model (Table S12). For the series of chloromethanes reacting with F_430_, the calculated activation barriers (Δ*G*_ET_^‡^) progressively decrease with increasing chlorine substitution (36.4 kcal/mol for CH_3_Cl; 31.4 kcal/mol for CH_2_Cl_2_; 26.5 kcal/mol for CHCl_3_; 20.9 kcal/mol for CCl_4_). This trend arises because a higher degree of chlorination lowers the σ*­(C–Cl) antibonding orbital energy, enhances the substrate’s electron affinity, and thereby increases the thermodynamic driving force (Δ*G*_ET_) collectively leading to a reduced electron transfer barrier (Δ*G*_ET_^‡^). In addition, the slightly larger molecular radius associated with higher chlorination diminishes the outer-sphere reorganization energy λ_O_, further contributing to the barrier reduction. Still, the overall driving force (Δ*G*_ET_) is positive of all investigated chloromethanes, being as high as 18.8 kcal/mol for CH_3_Cl. Although Δ*G*_ET_^‡^ decreases with increasing chlorine substitution, its absolute magnitude remains substantially greater than Δ*G* ^‡^ values calculated for the inner-sphere electron transfer and S_N_2 pathways (Figure 12). Therefore, from a kinetic standpoint, the dominance of the outer-sphere electron transfer mechanism can be excluded; the results indicate that the O-SET-D pathway is energetically disfavored, providing indirect support for the feasibility of the inner-sphere radical or S_N_2 mechanisms in halogen abstraction.

**Figure 12:**
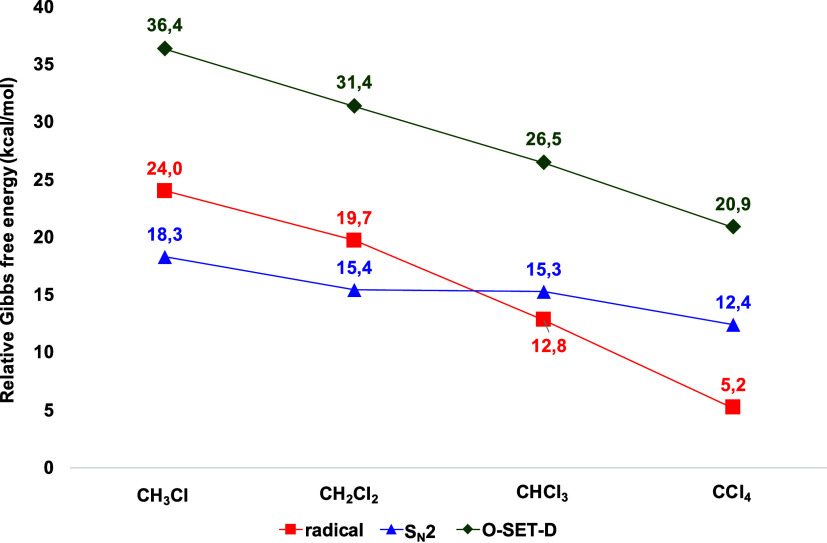
Dehalogenation of chloromethanes on F_430_: Δ*G* ^‡^ values for different mechanisms and substrates.

### The Mechanism Switch

The radical and S_N_2 surfaces cross between CH_2_Cl_2_ and CHCl_3_ (Figure 12); S_N_2 is lower for CH_3_Cl and CH_2_Cl_2_, but at CHCl_3_ the radical mechanism overtakes S_N_2, and the gap widens further for CCl_4_. Mechanistically, the radical TS is insulated from the severe steric constraints that plague S_N_2 inside F_430_ for CCl_4_: it does not require a close Ni–C approach and tolerates a bent geometry focused on Cl transfer.

Analogous trend can be seen for OEtiBCh. For the S_N_2 mechanism, all Δ*G* ^‡^ values are shifted downward by 2.0–3.7 kcal/mol compared to F_430_ (Figure S2 and Table S5); for the radical manifold, these differences rise to 3.9–6.1 kcal/mol (Figure S3 and Table S8). It is noteworthy that the OEtiBCh-catalyzed reactions are typically exergonic, as opposed to F_430_-catalyzed ones; however, the shape of mechanism switch plot (Figure S4) reproduces the same trend as for F_430_, clearly demonstrating the preference for the radical mechanism for CHCl_3_ and CCl_4_.

### Kinetic Isotope Effects (KIEs): Mechanistic Fingerprints across Substrates and Catalysts

We computed natural-abundance KIEs for all isotopically distinguishable positions of CH_3_Cl, CH_2_Cl_2_, CHCl_3_, and CCl_4_ for both pathways (Figure 13 and Tables S13–S14).

**Figure 13:**
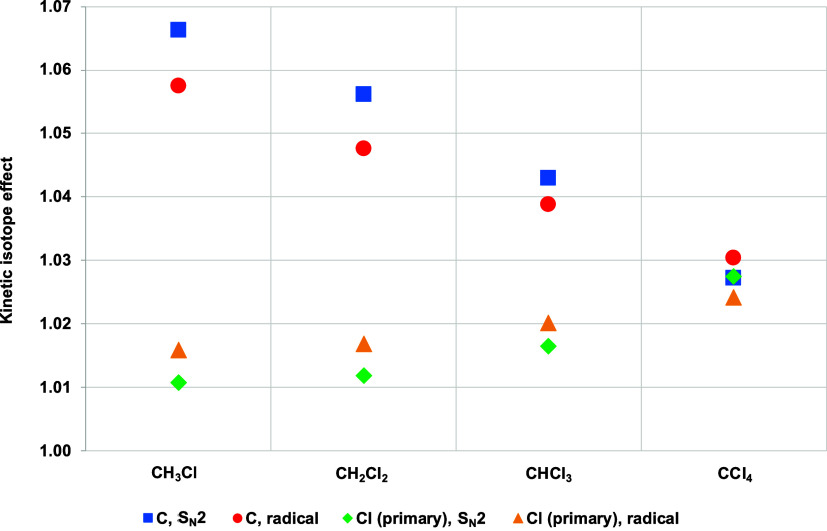
Kinetic isotopic effects for dehalogenation of chloromethanes on F_430_.

#### Primary ^13^C KIEs at the Reaction Center

For CH_3_Cl and CH_2_Cl_2_ the S_N_2 pathway exhibits a consistently larger ^13^C KIE than the radical pathway (e.g., CH_3_Cl: 1.066 vs 1.058), reflecting greater carbon-center reorganization along the S_N_2 coordinate. As chlorination increases, ^13^C values for both pathways compress toward unity (1.04 for CHCl_3_; 1.03 for CCl_4_), consistent with progressively “earlier at carbon” TSs. Catalyst identity has little effect for CH_3_Cl/CH_2_Cl_2_ and only modulates fine detail for CCl_4_, where both pathways are intrinsically subtle at carbon. ^37^Cl at the leaving group. Numerically, ^37^Cl KIEs are the smallest values measured (1.00–1.03), yet–because ^37^Cl comprises ca. 25% of natural chlorine–they exert the largest natural-abundance impact on the bulk rate constant among the probes considered. In practice, ^37^Cl remains a supporting discriminator: for CH_3_Cl the radical pathway tends to show slightly larger ^37^Cl than S_N_2, whereas for more highly chlorinated substrates the values converge and become less diagnostic.

#### Deuterium Secondary Isotope Effects

For CH_3_Cl and CH_2_Cl_2_ the radical pathway exhibits very large, normal β-D KIEs (1.27–1.33), clearly exceeding S_N_2 (typically 1.10–1.18). For CHCl_3_ (single β-D), S_N_2 presents an exceptionally large β-D effect (1.32–1.37), larger than radical (1.28–1.31), in line with a later, hyperconjugation-sensitive S_N_2 TS in this substrate class. As expected, hydrogen shows the numerically largest KIEs because the fractional mass change (H → D) is maximal and small vibrational/rehybridization differences translate into large zero-point shifts at β-C. Nevertheless, because deuterium is only ca. 0.016% at natural abundance, these large numerical KIEs have a negligible effect on the bulk rate of an unlabeled sample.

#### Secondary ^37^Cl (Nonleaving Chlorines) Isotope Effects

Across CH_2_Cl_2_, CHCl_3_, and CCl_4_ the nonleaving chlorines exhibit KIEs essentially at unity, typically 0.998–1.003 for both mechanisms. Very weakly inverse values in S_N_2 likely reflect slight angular compression at carbon that softens spectator C–Cl modes; conversely, the radical TS can produce a barely normal tilt from diffuse polarization. These effects are not mechanistically diagnostic and, although ^37^Cl is abundant, their contribution to the overall natural-abundance rate is much smaller than that of the leaving-chloride site.

Taken together, chlorinated derivatives of methane organize a clear diagnostic palette. For CH_3_Cl/CH_2_Cl_2_, the pairing “larger ^13^C + moderate β-D” (S_N_2) versus “smaller ^13^C + very large β-D” (radical) clearly distinguishes mechanisms; ^37^Cl provides secondary support. For CHCl_3_, the single β-D is the most informative (S_N_2 > radical). For CCl_4_, all KIEs are subtle and should be interpreted alongside barriers and TS geometries.

The KIE landscape aligns with the TS metrics and barrier trends. In S_N_2 pathways for CH_3_Cl and CH_2_Cl_2_, the carbon center partially planarizes in the TS (backside displacement with substantial C–Cl elongation), which amplifies the primary ^13^C KIE relative to the radical pathway, where carbon remains closer to tetrahedral and the incipient R^•^ forms with less carbon-centric reorganization. Conversely, the pronounced loss of β-C hyperconjugation in the radical pathway–and associated high-frequency C–H mode stiffening–drives its characteristically large β-D KIEs. For CHCl_3_ the S_N_2 TS is later with respect to C–Cl cleavage and β-hyperconjugation, explaining the exceptionally large single-H effect; the radical TS is earlier and correspondingly smaller in β-D.

Considering chlorine atoms, both mechanisms show relatively early motion of the leaving chlorine for CCl_4_ (hence small ^37^Cl), while for CH_3_Cl the radical TS places slightly more dynamical weight on Cl departure than S_N_2, inflating ^37^Cl modestly. Although the numerical magnitude of ^37^Cl KIEs is small, their practical influence on an unlabeled ensemble is the largest among the probes because ^37^Cl is abundant; in contrast, the visually striking β-D KIEs barely nudge bulk rates at natural abundance. Consistent with the TS metrics, secondary chlorines remain spectators: their C–Cl distances and local force constants change only subtly along either pathway, rationalizing near-unity secondary ^37^Cl KIEs and their negligible mechanistic leverage compared with the leaving-chloride site.

### Dual Slope Analysis

In the environmental studies dual-slope isotope analysis is a convenient tool^56,57^ used for identification of dehalogenation processes including dehalogenation of chloromethanes.^20,56,58,59^ In the field experiments usually only the remaining reactant can be subjected to isotopic analysis leading to the average isotopic composition of a given element (chlorine in our case); in laboratory experiments it is also possible to measure product isotopic composition which enables determination of the primary isotope effect. We have therefore calculated Λ­(C/Cl) for mechanisms and reactants considered in this study corresponding to both of these experimental procedures; the results are collected in Table 1.

**Table 1:** Λ­(C/Cl) Calculated for F_430_-Catalyzed Dehalogenations of Chloromethanes

mechanism	S_N_2		radical	
substrate	1° KIEs	avg. KIEs	1° KIEs	avg. KIEs
CH_3_Cl	5.77	5.77	3.28	3.28
CH_2_Cl_2_	4.40	9.66	2.74	5.20
CHCl_3_	2.50	8.39	1.87	5.25
CCl_4_	0.97	3.42	1.13	3.78

When Λ values are compared within a given substrate, a clear tendency is observed for CH_3_Cl, CH_2_Cl_2_, and CHCl_3_: Λ is consistently lower for the radical pathway than for the S_N_2 pathway. In this substrate-resolved sense, Λ can therefore provide mechanistic guidance. However, this ordering is not universal, as illustrated by CCl_4_ on F_430_, where Λ for the radical mechanism slightly exceeds the S_N_2 value (Table 1). This exception shows that Λ alone cannot be treated as an error-proof mechanistic discriminator across the entire chloromethane series.

A second important limitation is the dependence of Λ on the experimental observable (primary vs average). For a fixed mechanism, Λ derived from primary KIEs decreases systematically with increasing chlorination, consistent with the progressive lowering of carbon isotope effects. In contrast, Λ derived from average KIEs exhibits a less regular, sometimes nonmonotonic behavior because bulk isotope signatures are influenced by site averaging and by the inclusion of nonreacting positions in the isotopic observable. Consequently, mechanistic assignments based solely on Λ become contingent on both the specific substrate and the measurement protocol, and Λ should be interpreted as a process indicator rather than a transferable mechanistic fingerprint.

## Conclusions

Across the entire data set, a coherent picture emerges of how F_430_ negotiates between the two mechanistic manifolds. Three physical levers control where the radical surface eclipses the S_N_2 surface: electrostatics, sterics, and orientation along the C–Cl axis.

Electrostatically, the key distinction is how well the pocket can stabilize charge separation as the C–Cl bond is polarized. Along the S_N_2 surface, especially for the more highly chlorinated substrates, the transition state and the post-TS ion-pair C2 feature substantial positive charge on the carbon fragment and negative charge on the departing chloride. For CH_3_Cl and CH_2_Cl_2_ this charge separation is moderate and can be comfortably accommodated by the neutral macrocycle in a polar medium, leading to barriers that are competitive with the radical pathway. For CCl_4_, however, heterolytic cleavage creates a strongly cationic CCl_3_ fragment that is only weakly stabilized by F_430_. The C2 basin remains shallow and the S_N_2 barrier rebounds, even though the TS geometry shows a nearly linear, Cl-bridged arrangement with Ni–C distances close to those in C2. In contrast, along the radical surface the charge is more evenly distributed between Ni and Cl in the compact Ni–Cl forming TS, so electrostatic penalties are smaller and the barrier continues to decrease with chlorination.

Steric effects then modulate how these electrostatic preferences are expressed. The densely substituted F_430_ pocket channels the approach of the substrate and limits how closely Ni can reach the electrophilic carbon when the substrate is large and heavily chlorinated. For CH_3_Cl and CH_2_Cl_2_, a relatively compact, partially backside S_N_2 TS can still be realized. For CHCl_3_ and especially CCl_4_, the same steric constraints force the S_N_2 TS to be geometrically loose and strongly Cl-bridged, with long Ni–C distances and low |ν_im_|. In contrast, the radical TS does not require direct Ni–C engagement and remains compact around the Ni–Cl coordinate even for CCl_4_, leaving its barrier comparatively insensitive to pocket crowding. This difference explains why the radical and S_N_2 barriers cross between CH_2_Cl_2_ and CHCl_3_ and why the radical pathway becomes overwhelmingly favored for CCl_4_.

Orientation provides a finer level of control, particularly for the intermediate chlorination states. For CH_2_Cl_2_ and CHCl_3_, rotating the substrate about the C–Cl bond alters the alignment between the incoming Ni vector and the σ*­(C–Cl) orbital. On the radical surface, good alignment yields a well-focused halogen-atom-transfer TS with efficient flow into σ*­(C–Cl) and a noticeably lower barrier; misalignment produces a looser TS and raises ΔG^‡^ by up to about a kilocalorie without changing the macrocycle conformer. On the S_N_2 surface, orientation similarly tunes how much of the reaction coordinate is expressed as Ni–C approach versus Cl departure. In this sense, the mechanism switch is not determined solely by global barrier heights, but also by how easily the pocket can steer σ*­(C–Cl) into a productive direction for each pathway.

From the perspective of using isolated F_430_ as the dehalogenation catalyst in soils one other aspect is the protonation of the carboxylic groups which can vary with the soil pH. On the example of S_N_2 methyl chloride dehalogenation we have shown that deprotonation of all five groups has minor effect on geometries (see Tables S3 and S6 in Supporting Information) and the corresponding Gibbs free energy of activation diminishes by only 1.7 kcal/mol which might indicate that the dehalogenation processes in basic soils proceed slightly faster than in the acidic ones.

Computed isotope signatures support the mechanistic picture but also reveal the limits of dual-isotope diagnostics. Across the series, primary ^13^C and ^37^Cl KIEs progressively compress toward unity with increasing chlorination, consistent with reduced carbon-centered rehybridization and the growing importance of leaving-group-centered reorganization. Secondary β-D effects remain comparatively sensitive and therefore provide an additional handle for pathway discrimination where primary isotope effects become small. In contrast, dual-element slopes Λ are substrate- and protocol-dependent: within individual substrates Λ is generally lower for the radical pathway than for S_N_2, yet the ordering can break down in the most highly chlorinated limit (CCl_4_ on F_430_), and Λ derived from average KIEs can behave nonmonotonically due to site-averaging. Consequently, Λ should be treated as a process indicator rather than a transferable mechanistic fingerprint.

From a design perspective, OEtiBCh serves as a useful minimal surrogate for F_430_. Once the global S conformer and the dominant substrate approach geometry are identified, the simplified fragment reproduces the qualitative trend seen in the full cofactor: the same crossover from S_N_2 to radical with increasing chlorination, similar evolution of Ni–C/Ni–Cl/C–Cl metrics along the series, and comparable KIE patterns. The main quantitative differences are systematic: barriers are typically several kcal/mol lower and C2/P wells are more exergonic in OEtiBCh than in F_430_. This suggests that OEtiBCh-level models can be employed for rapid mechanistic screening and for exploratory work on new substrates, with the understanding that absolute barriers will be shifted upward and equilibria less product-favored in the real macrocycle. Notably, finite explicit-solvent (DMF) clusters did not improve mechanistic trends or barrier predictions relative to the implicit SMD treatment, and in several cases introduced additional variability attributable to local solvent configurations rather than chemistry of the reactive coordinate.

## Supplementary Material



## Notes

All numerical results for F_430_-catalyzed reactions and graphical and numerical results for OEtiBCh-catalyzed reactions (PDF)
