# Supplementary material for: Mechanism of F430-Catalyzed Dehalogenations of Chloromethanes: A DFT Perspective
Source: J Phys Chem B. 2026 Apr 20;130(17):4547–59. doi: 10.1021/acs.jpcb.6c00679 (PMC13298895; doi:10.1021/acs.jpcb.6c00679)
Supplement: Supplementary file 1 [file jp6c00679_si_001.pdf]

Supporting Information

Mechanism of F_{430}-Catalyzed Dehalogenations of Chloromethanes. A DFT Perspective

Ye Han,[1] Mateusz Nowicki,[1] Mateusz Pokora,[1,2] Li Ji,[3] Piotr Paneth[1]

[1] International Center for Research on Innovative Biobased Materials (ICRI-BioM)—International Research Agenda, Lodz University of Technology, Zeromskiego 116, Lodz 90–924, Poland

[2] Centre of Molecular and Macromolecular Studies, Polish Academy of Sciences, Sienkiewicza 112, 90-363 Lodz, Poland

[3] School of Environment and Spatial Informatics, China University of Mining and Technology, Xuzhou 221116, China

Table S1. S_N2 dehalogenation of CH_3Cl on F430: Gibbs free energies of stationary points for the 10 conformers with lowest $\Delta G^‡$.

Conformer	F430	MeCl	C1	TS	C2	$[F430–Me]^+$	Cl^-
**1**	-4443.995261	-500.031312	-4944.023341	-4943.998816	-4944.009384	-4483.664954	-460.345015
**2**	-4443.988910	-500.031312	-4944.016109	-4943.991073	-4943.999342	-4483.656890	-460.345015
**5**	-4443.987918	-500.031312	-4944.016893	-4943.994108	-4944.006279	-4483.657039	-460.345015
**10**	-4443.982293	-500.031312	-4944.013392	-4943.990646	-4944.003875	-4483.657923	-460.345015
**17**	-4443.989546	-500.031312	-4944.017563	-4943.992478	-4944.003495	-4483.662349	-460.345015
**20**	-4443.995029	-500.031312	-4944.022075	-4943.996672	-4944.004699	–	-460.345015
**23**	-4443.988617	-500.031312	-4944.017433	-4943.993702	-4944.003853	-4483.658618	-460.345015
**25**	-4443.983421	-500.031312	-4944.014132	-4943.992490	-4944.004173	-4483.657176	-460.345015
**26**	-4443.987495	-500.031312	-4944.016053	-4943.994929	-4944.006593	-4483.659732	-460.345015
**27**	-4443.998948	-500.031312	-4944.027838	-4944.001090	-4944.008055	-4483.666369	-460.345015

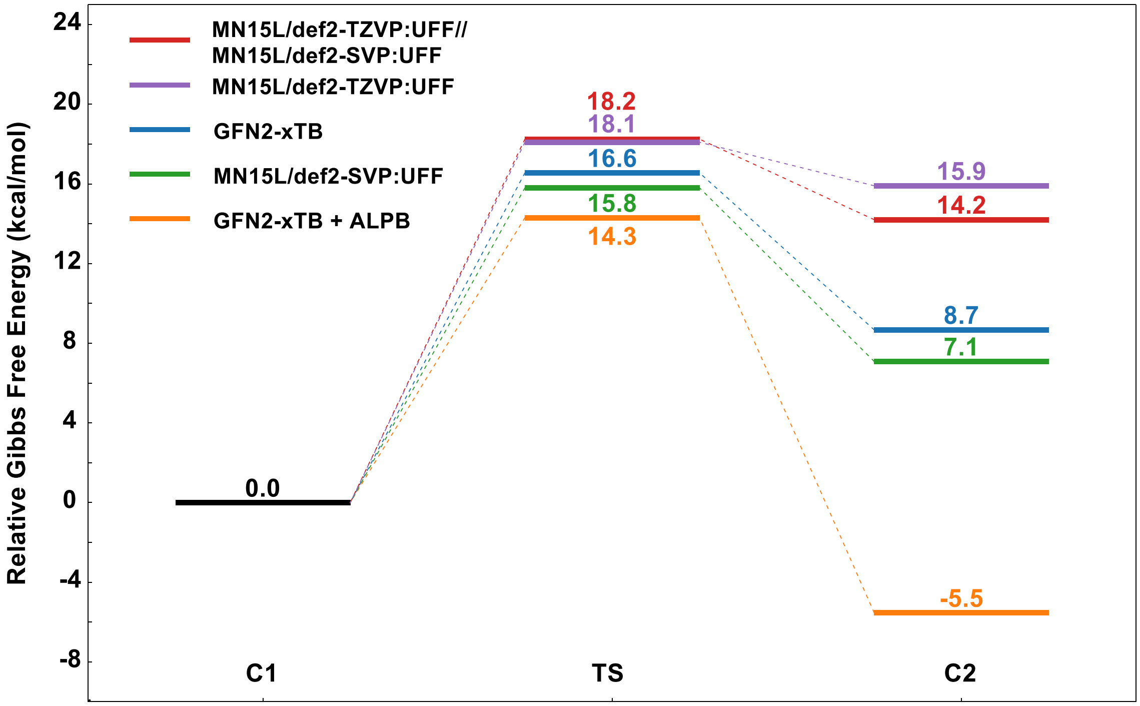

Figure S1. S_N2 dehalogenation of CH_3Cl on OEtiBCh: Gibbs free energy profiles in a cluster of 54 DMF molecules.

Table S2. S_N2 dehalogenation of CH_3Cl on OEtiBCh in a cluster of 54 DMF molecules: Gibbs free energies of stationary points.

Method	Basis set	Implicit solvation	C1	TS	C2
GFN2-xTB	–	–	-1038.961945	-1038.935561	-1038.948119
GFN2-xTB	–	ALPB	-1039.292079	-1039.269283	-1039.300901
MN15L:UFF	def2-SVP	–	-3618.447093	-3618.421909	-3618.435798
MN15L:UFF	def2-TZVP//def2-SVP	–	-3620.953325	-3620.924243	-3620.930684
MN15L:UFF	def2-TZVP	–	-3620.958576	-3620.929744	-3620.933226

Table S3. S_N2 dehalogenation of CH_3Cl on OEtiBCh in SMD-modeled water, soil and DMF: Gibbs free energies of stationary points.

Solvent	OEtiBCh	MeCl	C1	TS	C2	OEtiBCh–Me	Cl^-
**Water**[a]	-4444.003876	–	-4941.777044	-4941.754697	–	–	–
**Water**	-4444.003876	-500.027995	-4944.029971	-4944.003775	-4944.010967	-4483.668080	-460.344039
**Soil**	-4443.999706	-500.031343	-4944.027775	-4943.999992	-4944.004616	-4483.660972	-460.341015
**DMF**	-4443.998900	-500.031312	-4944.028452	-4944.001090	-4944.008082	-4483.666378	-460.345015

[a] All carboxylic groups of F430 were deprotonated.

Table S4. S_N2 dehalogenation of chloromethanes on F430: Gibbs free energies of stationary points.

Substrate	Conformer	F430	R–Cl	C1	TS	C2	$[F430–R]^+$	Cl^-
CH_3Cl	**1**[a]	-4443.998900	-500.031312	-4944.028452	-4944.001090	-4944.008082	-4483.666378	-460.345015
CH_3Cl	**2**	-4443.995261	-500.031312	-4944.023341	-4943.998816	-4944.009384	-4483.666352	-460.345015
CH_2Cl_2	**1a**	-4443.998900	-959.613309	-5403.613303	-5403.587629	-5403.601517	-4943.258900	-460.345015
CH_2Cl_2	**1b**	-4443.998900	-959.613309	-5403.609711	-5403.582769	-5403.602530	-4943.258900	-460.345015
CH_2Cl_2	**1c**	-4443.998900	-959.613309	-5403.613294	-5403.584557	-5403.604020	-4943.258900	-460.345015
CH_2Cl_2	**2a**	-4443.995261	-959.613309	-5403.607673	-5403.582832	-5403.611210	-4943.256526	-460.345015

**$CHCl_3$**	**1a**	-4443.998900	-1419.190410	-5863.191273	-5863.160957	-5863.185310	-5402.840572	-460.345015
**$CHCl_3$**	**1b**	-4443.998900	-1419.190410	-5863.191373	-5863.164140	-5863.196242	-5402.840572	-460.345015
**$CHCl_3$**	**1c**	-4443.998900	-1419.190410	-5863.193272	-5863.157614	-5863.191593	-5402.840572	-460.345015
**$CHCl_3$**	**2a**	-4443.995261	-1419.190410	-5863.187856	-5863.164932	-5863.185941	-5402.840572	-460.345015
**CCl_4**	**1**	-4443.998900	-1878.760194	-6322.757436	-6322.739327	-6322.740362	-5862.415826	-460.345015
**CCl_4**	**2**	-4443.995261	-1878.760194	-6322.754782	-6322.729147	-6322.744872	-5862.408258	-460.345015

a Numbers denote different arrangements of side chains within F430. Letters denote different rotations of R-Cl in transition state (rotation about C-Cl bond).

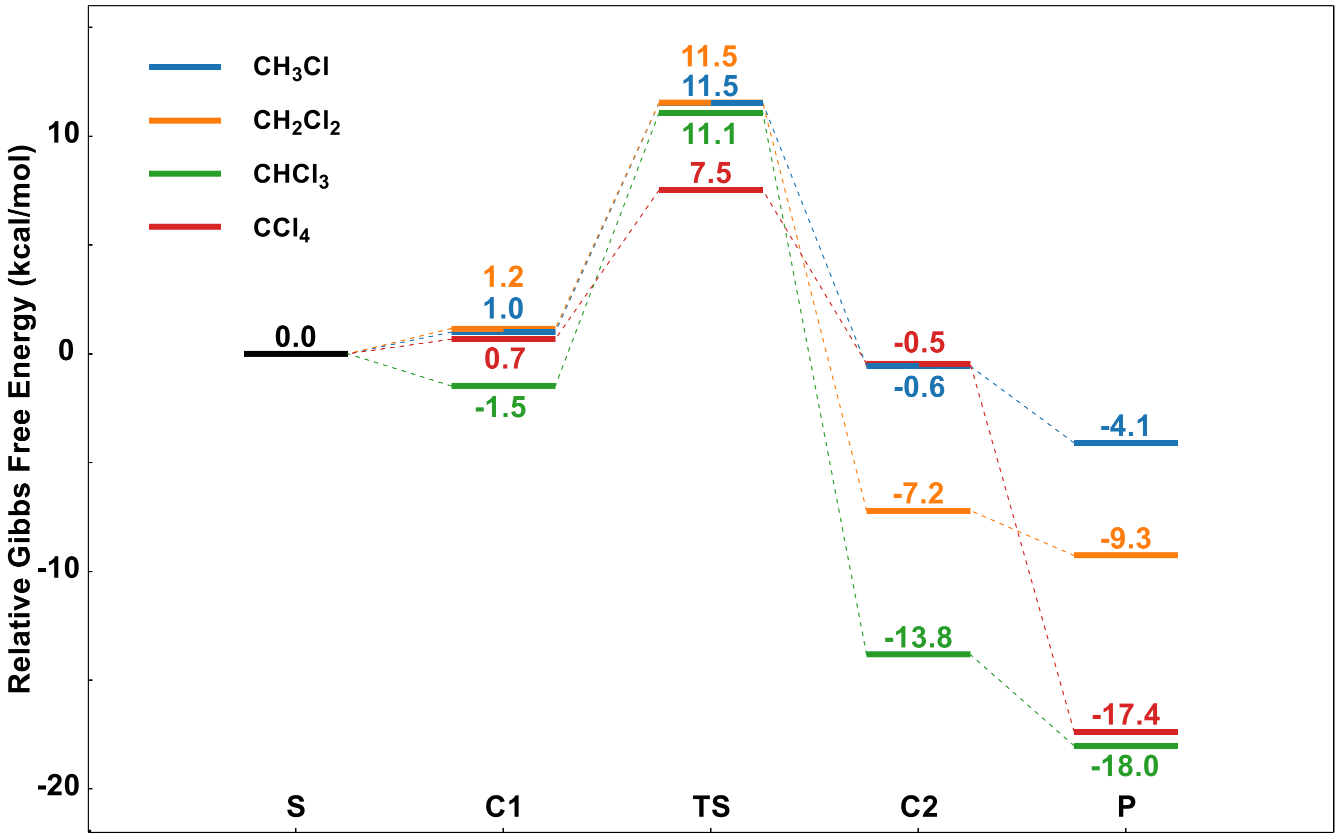

Figure S2. S_N2 dehalogenation of chloromethanes on OEtiBCh: Gibbs free energy profiles.

Table S5. S_N2 dehalogenation of chloromethanes on OEtiBCh: Gibbs free energies of stationary points.

Substrate	OEtiBCh	R–Cl	C1	TS	C2	OEtiBCh–R	Cl^-
**CH_3Cl**	-3126.729654	-500.031312	-3626.758336	-3626.741567	-3626.760834	-3166.421437	-460.345015
**CH_2Cl_2**	-3126.729654	-959.613309	-4086.341109	-4086.324580	-4086.354446	-3626.012728	-460.345015
**$CHCl_3$**	-3126.729654	-1419.190410	-4545.922420	-4545.902419	-4545.942097	-4085.603759	-460.345015
**CCl_4**	-3126.729654	-1878.760194	-5005.488780	-5005.477859	-5005.490576	-4545.172516	-460.345015

Table S6. S_N2 dehalogenation of chloromethanes: geometric parameters of transition states.

Cofactor	Substrate	Solvent	$\|\nu_{im}\|$	Ni–C	C–Cl	Ni–C–Cl
F430	CH_3Cl	soil	340.91	2.29	2.40	175.84
F430	CH_3Cl	water	363.42	2.32	2.37	177.27
F430[a]	CH_3Cl	water	377.46	2.36	2.33	174.17
F430	CH_3Cl	DMF	354.18	2.32	2.38	174.63
F430	CH_2Cl_2	DMF	363.68	2.48	2.37	148.53
F430	$CHCl_3$	DMF	294.97	2.80	2.43	136.05
F430	CCl_4	DMF	161.44	3.93	2.36	139.02
OEtiBCh	CH_3Cl	DMF	404.31	2.44	2.27	179.01
OEtiBCh	CH_2Cl_2	DMF	397.89	2.58	2.27	152.22
OEtiBCh	$CHCl_3$	DMF	303.80	2.98	2.34	141.32
OEtiBCh	CCl_4	DMF	303.36	4.04	2.12	142.86

[a] All carboxylic groups of F430 were deprotonated.

Table S7. Radical dehalogenation of chloromethanes on F430: Gibbs free energies of stationary points.

Substrate	Conformer	F430	R–Cl	C1	TS	C2	F430–Cl	R•
**CH_3Cl**	1	-4443.998900	-500.031312	-4944.030212	-4944.023240	-4943.991922	-4944.005429	-4944.006507
**CH_3Cl**	2	-4443.995261	-500.031312	-4944.026573	-4944.018590	-4943.988483	-4944.006217	-4944.008501
**CH_3Cl**	3	-4443.987495	-500.031312	-4944.018807	-4944.013780	-4943.982893	-4944.001220	-4944.007235
**CH_2Cl_2**	1a	-4443.998900	-959.613309	-5403.612209	-5403.611381	-5403.580444	-5403.592396	-5403.596849
**CH_2Cl_2**	1b	-4443.998900	-959.613309	-5403.612209	–	-5403.580754	–	-5403.596849
**CH_2Cl_2**	1c	-4443.998900	-959.613309	-5403.612209	-5403.609991	-5403.580745	-5403.597365	-5403.596849
**CH_2Cl_2**	2a	-4443.995261	-959.613309	-5403.608570	-5403.603232	-5403.578407	-5403.600354	-5403.598843
**CH_2Cl_2**	2b	-4443.995261	-959.613309	-5403.608570	-5403.603246	-5403.576512	-5403.590619	-5403.598843
**CH_2Cl_2**	2c	-4443.995261	-959.613309	-5403.608570	-5403.599820	-5403.578239	-5403.597566	-5403.598843
**CH_2Cl_2**	3a	-4443.987495	-959.613309	-5403.600804	-5403.598772	-5403.573027	-5403.588577	-5403.597577
**CH_2Cl_2**	3b	-4443.987495	-959.613309	-5403.600804	-5403.595790	-5403.572652	-5403.584313	-5403.597577
**CH_2Cl_2**	3c	-4443.987495	-959.613309	-5403.600804	-5403.594995	-5403.573200	-5403.592703	-5403.597577
**$CHCl_3$**	1a	-4443.998900	-1419.190410	-5863.189310	-5863.185095	-5863.168255	-5863.182486	-5863.183693
**$CHCl_3$**	1b	-4443.998900	-1419.190410	-5863.189310	-5863.186523	-5863.168863	-5863.180551	-5863.183693
**$CHCl_3$**	1c	-4443.998900	-1419.190410	-5863.189310	-5863.186477	-5863.168839	-5863.180579	-5863.183693
**$CHCl_3$**	2a	-4443.995261	-1419.190410	-5863.185671	-5863.179654	-5863.167077	–	-5863.185687
**$CHCl_3$**	2b	-4443.995261	-1419.190410	-5863.185671	-5863.179617	-5863.167135	-5863.181935	-5863.185687
**$CHCl_3$**	2c	-4443.995261	-1419.190410	-5863.185671	–	-5863.167474	-5863.178946	-5863.185687
**$CHCl_3$**	3a	-4443.987495	-1419.190410	-5863.177905	-5863.173699	-5863.162203	-5863.177287	-5863.184421
**$CHCl_3$**	3b	-4443.987495	-1419.190410	-5863.177905	-5863.175886	-5863.162283	-5863.175857	-5863.184421
**$CHCl_3$**	3c	-4443.987495	-1419.190410	-5863.177905	-5863.174660	-5863.162097	-5863.178292	-5863.184421
**CCl_4**	1	-4443.998900	-1878.760194	-6322.759094	-6322.757400	-6322.750800	-6322.764700	-6322.765222
**CCl_4**	2	-4443.995261	-1878.760194	-6322.755455	-6322.753808	-6322.750131	-6322.765193	-6322.767216
**CCl_4**	3	-4443.987495	-1878.760194	-6322.747689	-6322.749860	-6322.744666	-6322.760481	-6322.765950

a Numbers denote different arrangements of side chains within F430. Letters denote different rotations of R-Cl in transition state (rotation about C-Cl bond).

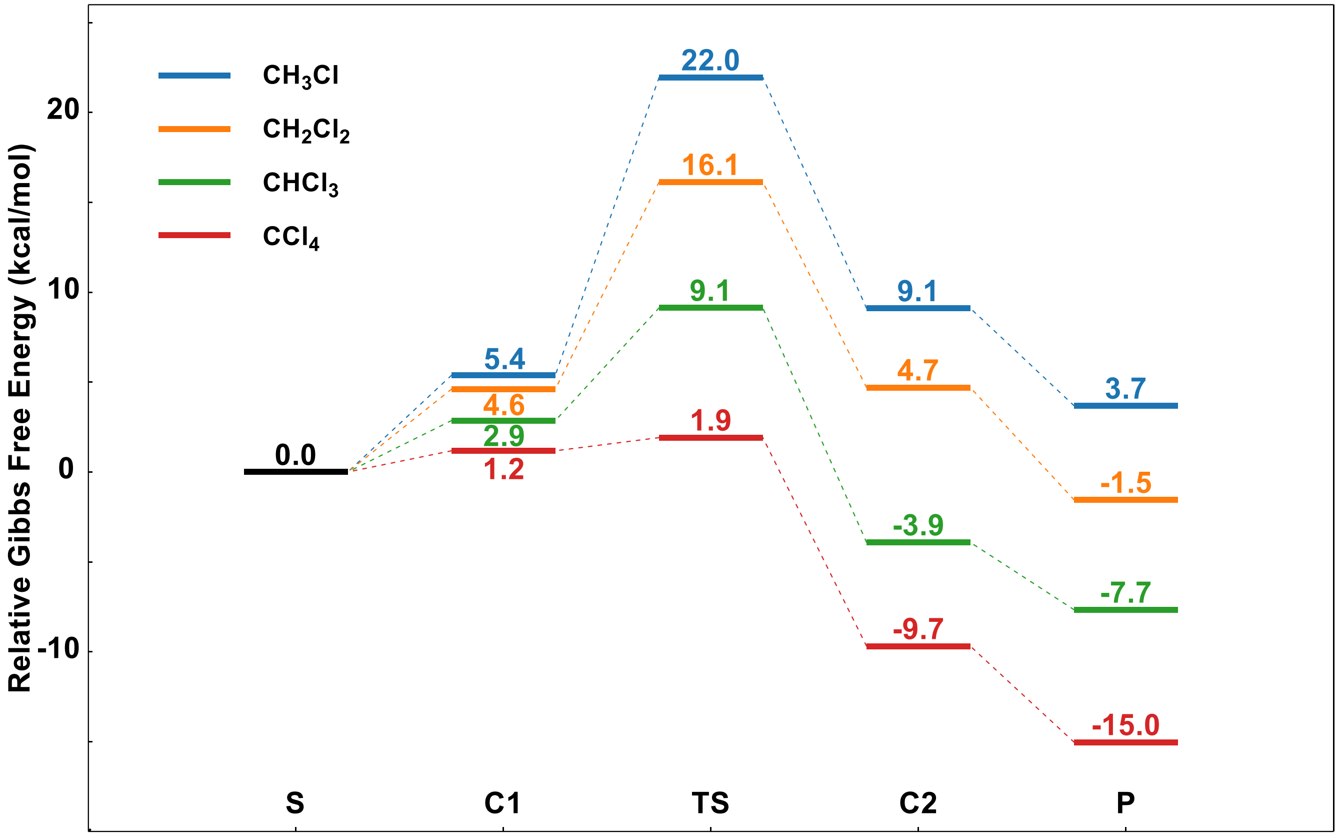

Figure S3. Radical dehalogenation of chloromethanes on OEtiBCh: Gibbs free energy profiles.

Table S8. Radical dehalogenation of chloromethanes on OEtiBCh: Gibbs free energies of stationary points.

Substrate	OEtiBCh	R–Cl	C1	TS	C2	OEtiBCh–Cl	R•
CH_3Cl	-3126.729654	-500.031312	-3626.752385	-3626.725980	-3626.746451	-3586.964229	-39.790862
CH_2Cl_2	-3126.729654	-959.613309	-4086.335612	-4086.317254	-4086.335475	-3586.964229	-499.381204
$CHCl_3$	-3126.729654	-1419.190410	-4545.915504	-4545.905512	-4545.926307	-3586.964229	-958.968048
CCl_4	-3126.729654	-1878.760194	-5005.487966	-5005.486798	-5005.505290	-3586.964229	-1418.549577

Table S9. Radical dehalogenation of chloromethanes: geometric parameters of transition states.

**Cofactor**	**Substrate**	$\lvert\nu_{im}\rvert$	**Ni–Cl**	**Cl–C**	**Ni–Cl–C**
OEtiBCh	CH_3Cl	392.01	2.53	2.35	165.51
OEtiBCh	CH_2Cl_2	330.18	2.52	2.30	167.92
OEtiBCh	$CHCl_3$	287.38	2.52	2.21	167.03
OEtiBCh	CCl_4	181.56	2.54	2.03	169.08
F430	CH_3Cl	342.93	2.46	2.45	177.40
F430	CH_2Cl_2	301.12	2.45	2.40	158.46
F430	$CHCl_3$	297.59	2.46	2.29	154.33
F430	CCl_4	241.94	2.45	2.18	158.22

Table S10. SN2 dehalogenation of chloromethanes on F430: Merz-Singh-Kollman charges.

**Substrate**	**Moiety**	**C1**	**TS**	**C2**
**CH_3Cl**	$-CH_3$	0.193929	0.183653	-0.038544
**CH_2Cl_2**	$-CH_2Cl$	0.145203	0.384091	0.249594
**$CHCl_3$**	$-CHCl_2$	-0.341960	-0.019648	-0.093029
**CCl_4**	$-CCl_3$	-0.151055	0.136026	0.227197
**CH_3Cl**	-Cl	-0.193513	-0.640677	-0.891780
**CH_2Cl_2**	-Cl	-0.049282	-0.580876	-0.866693
**$CHCl_3$**	-Cl	0.059080	-0.552974	-0.904029
**CCl_4**	-Cl	0.114832	-0.547229	-0.872025

Table S11. Radical dehalogenation of chloromethanes on F430: Merz-Singh-Kollman charges.

Substrate	Moiety	C1	TS	C2
**CH_3Cl**	-CH_3	0.218686	0.034532	0.004484
**CH_2Cl_2**	-CH_2Cl	0.146925	0.024838	-0.011875
**$CHCl_3$**	-$CHCl_2$	0.000044	-0.026008	-0.026728
**CCl_4**	-CCl_3	-0.178037	-0.171372	-0.045168
**CH_3Cl**	-Cl	-0.202247	-0.237723	-0.860965
**CH_2Cl_2**	-Cl	-0.216437	-0.403528	-0.524678
**$CHCl_3$**	-Cl	-0.061033	-0.357909	-0.551960
**CCl_4**	-Cl	0.142425	-0.156630	-0.498219

Table S12. Free energy barriers (kcal/mol) of the outer-sphere dissociative single-electron transfer ($\Delta G^{\ddagger}_{ET}$) in F430.

Substrate	ΔG_{ET}	λ_O	$\Delta G^{\neq}_{ET}$
**CH_3Cl**	18.8	104.9	36.4
**CH_2Cl_2**	13.5	96.8	31.4
**$CHCl_3$**	7.4	90.7	26.5
**CCl_4**	0.1	83.4	20.9

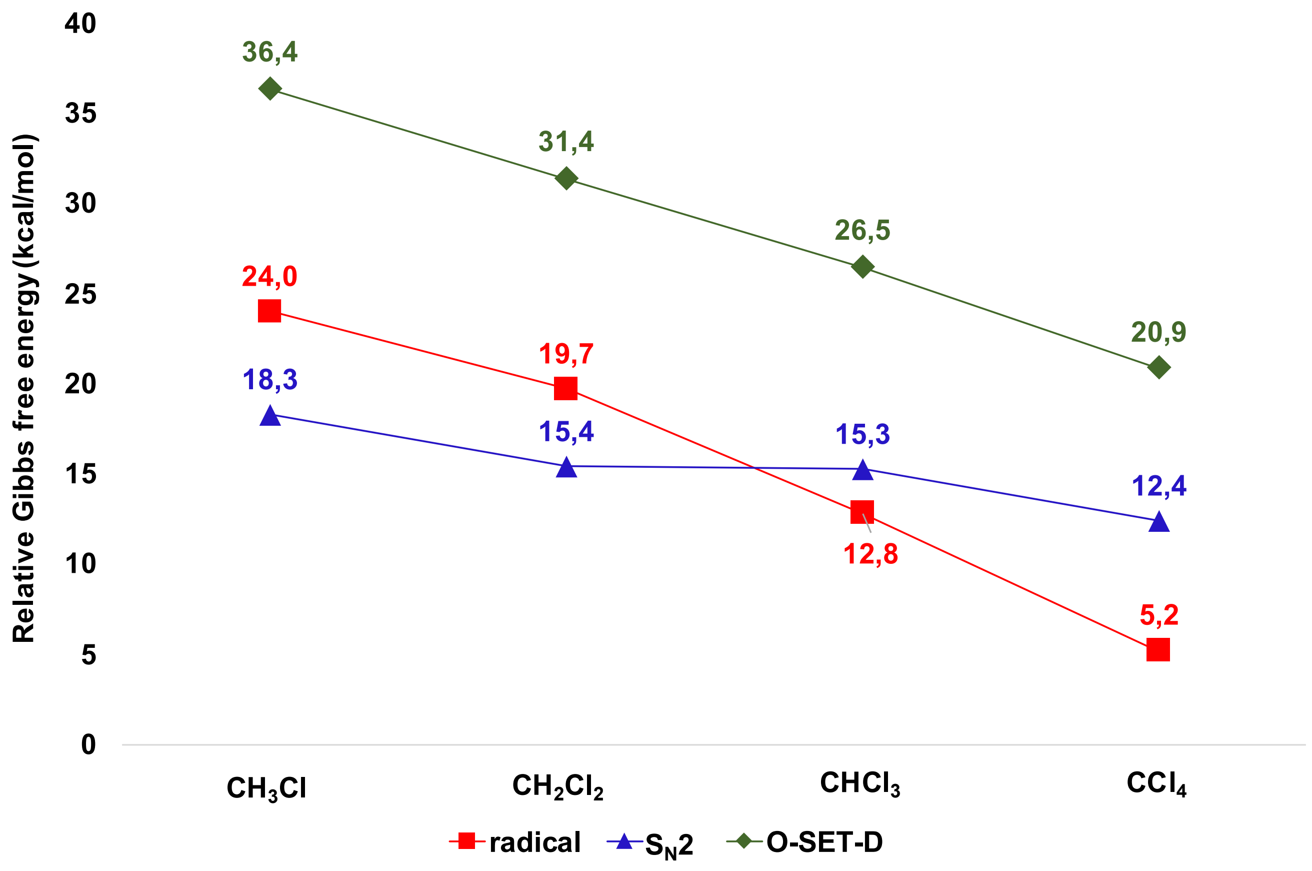

Figure S4. Dehalogenation of chloromethanes on OEtiBCh: $\Delta G^{\ddagger}$ values for different mechanisms and substrates.

Table S13. Dehalogenation of chloromethanes: kinetic isotope effects calculated on transition states and separate substrates.

Substrate	Cofactor	Mechanism	C	Cl <pr>	Cl (2)	Cl (3)	Cl (4)	H (1)	H (2)	H (3)
CH_3Cl	OEtiBCh	S_N2	1.065748	1.010716	–	–	–	1.089834	1.135924	1.101128
CH_3Cl	OEtiBCh	radical	1.057652	1.015701	–	–	–	1.312897	1.285091	1.296776
CH_3Cl	F430	S_N2	1.066300	1.010669	–	–	–	1.146160	1.090385	1.104812
CH_3Cl	F430	radical	1.057526	1.015786	–	–	–	1.314263	1.303535	1.312426
CH_2Cl_2	OEtiBCh	S_N2	1.057305	1.012580	0.998910	–	–	1.118047	1.185993	–
CH_2Cl_2	OEtiBCh	radical	1.048734	1.017308	1.000749	–	–	1.306576	1.296681	–

**CH_2Cl_2**	F430	S_N2	1.056135	1.011757	0.998714	–	–	1.127138	1.195791	–
**CH_2Cl_2**	F430	radical	1.047604	1.016844	0.999610	–	–	1.335378	1.321683	–
**$CHCl_3$**	OEtiBCh	S_N2	1.045006	1.018393	0.999767	0.999245	–	1.315882	–	–
**$CHCl_3$**	OEtiBCh	radical	1.040326	1.021321	1.001032	1.001383	–	1.249962	–	–
**$CHCl_3$**	F430	S_N2	1.043007	1.016478	0.998218	0.998946	–	1.370316	–	–
**$CHCl_3$**	F430	radical	1.038808	1.020064	1.000773	0.999707	–	1.291814	–	–
**CCl_4**	OEtiBCh	S_N2	1.039234	1.025100	1.002561	1.001616	1.001844	–	–	–
**CCl_4**	OEtiBCh	radical	1.027020	1.026389	1.002834	1.001405	1.002473	–	–	–
**CCl_4**	F430	S_N2	1.027175	1.027458	1.002133	1.000647	1.000996	–	–	–
**CCl_4**	F430	radical	1.030396	1.024198	1.000469	1.001820	1.002111	–	–	–

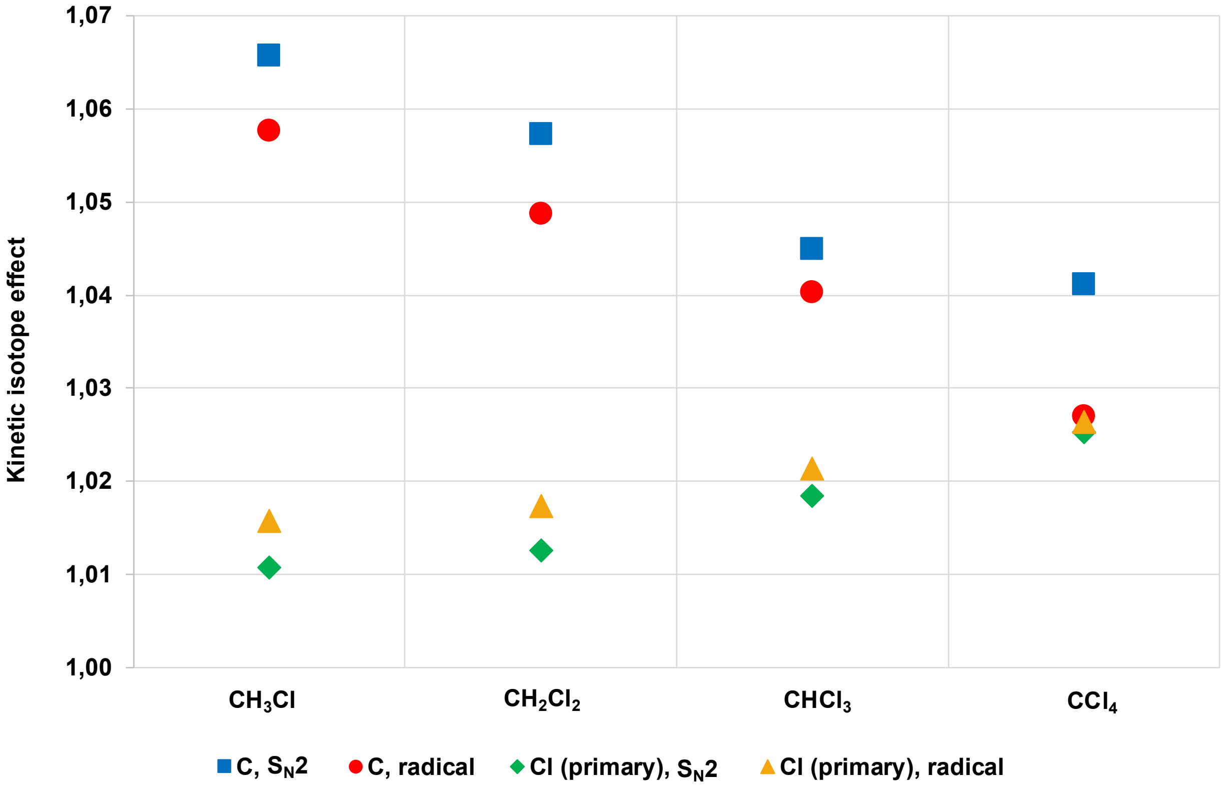

Figure S5. Kinetic isotopic effects for dehalogenation of chloromethanes on OEtiBCh.

Table S14. Dehalogenation of chloromethanes: kinetic isotope effects calculated on transition states and encounter complex.

Substrate	Cofactor	Mechanism	C	Cl <pr>	Cl (2)	Cl (3)	Cl (4)	H (1)	H (2)	H (3)
**CH_3Cl**	OEtiBCh	S_N2	1.063189	1.010345	–	–	–	1.139049	1.073336	1.101963
**CH_3Cl**	OEtiBCh	radical	1.057529	1.016149	–	–	–	1.298044	1.270021	1.275797
**CH_3Cl**	F430	S_N2	1.065605	1.010596	–	–	–	1.127792	1.118913	1.103993
**CH_3Cl**	F430	radical	1.057695	1.016504	–	–	–	1.310526	1.311428	1.306412
**CH_2Cl_2**	OEtiBCh	S_N2	1.056570	1.012741	0.999391	–	–	1.101447	1.156523	–
**CH_2Cl_2**	OEtiBCh	radical	1.048701	1.017814	1.000798	–	–	1.277669	1.271190	–
**CH_2Cl_2**	F430	S_N2	1.055720	1.011735	0.998948	–	–	1.170997	1.182324	–
**CH_2Cl_2**	F430	radical	1.048271	1.017210	1.000568	–	–	1.319292	1.325393	–
**$CHCl_3$**	OEtiBCh	S_N2	1.042520	1.018355	0.999998	0.999281	–	1.231784	–	–
**$CHCl_3$**	OEtiBCh	radical	1.038249	1.021347	1.001172	1.001181	–	1.235408	–	–
**$CHCl_3$**	F430	S_N2	1.041191	1.016275	0.998905	0.998977	–	1.272203	–	–
**$CHCl_3$**	F430	radical	1.038051	1.019951	1.000636	1.000493	–	1.262821	–	–
**CCl_4**	OEtiBCh	S_N2	1.036903	1.024738	1.002625	1.001863	1.001707	–	–	–
**CCl_4**	OEtiBCh	radical	1.018580	1.024364	1.002078	1.001093	1.001483	–	–	–
**CCl_4**	F430	S_N2	1.022893	1.026918	1.001609	0.999137	1.001618	–	–	–
**CCl_4**	F430	radical	1.026100	1.022703	1.001051	1.001359	1.001585	–	–	–

Table S15. Λ(C/Cl) calculated for OEtiBCh-catalyzed dehalogenations of chloromethanes.

**Mechanism**	**S_N2**		**radical**	
**Substrate**	**1° KIE**	**avg. KIE**	**1° KIE**	**avg. KIE**
**CH_3Cl**	5.99	5.99	3.48	3.48
**CH_2Cl_2**	4.20	9.06	2.64	4.96
**$CHCl_3$**	2.33	7.32	1.78	4.81
**CCl_4**	1.39	4.40	0.80	2.68
